# Urban morphology and climate vulnerability assessment in Kuwait: A spatio-temporal predictive analysis utilizing deep neural network-enhanced markov chain models for 2050 and 2100

**DOI:** 10.1371/journal.pone.0318604

**Published:** 2025-08-18

**Authors:** Walid Al-Shaar, Xavier Lehmann, Noha Saad, Gremina Elmazi, Mohamad Al-Shaar, Christelle Tohme

**Affiliations:** 1 College of Engineering and Technology, American University of the Middle East, Egaila, Kuwait; 2 LVMT (Laboratoire Ville-Mobilité-Transport), Unité commune Université Gustave Eiffel and École des Ponts, Champs-sur-Marne, Paris, France; 3 Geography Department, CREEMO (Centre de Recherche en Environnement-Espace Méditer-ranée Orientale), Saint-Joseph University of Beirut, Campus des Sciences Humaines, Rue de Damas, Mar Mikhael, Beirut, Lebanon; University 20 Aout 1955 skikda, Algeria, ALGERIA

## Abstract

Rapid urban growth in Kuwait creates challenges for adapting to climate change. This study investigates the spatio-temporal dynamics of urban growth in Kuwait and assesses its climate change vulnerability using a Multi-Layer Perceptron Markov Chain Model (MLPMCM) to forecast land use and land cover (LULC) changes for the years 2050 and 2100. Utilizing historical LULC data from 1985, 2005, and 2022, along with various spatial drivers, the research predicts urban expansion patterns for 2050 and 2100. The model achieved high accuracy in predictions, indicating that proximity to coastlines, road networks, and commercial areas are the primary drivers of urban growth in Kuwait. The study projects significant urban expansion, particularly in North-Northwestern and South-Southwestern regions, with urban areas expected to increase from 819 km² in 2022–1,893 km² by 2100. Climate vulnerability analysis, based on RCP 8.5 scenario projections, is assessed using the cross-referencing approach and it suggests temperature increases of up to 17°C in urban and coastal regions by 2100. The research highlights the complex interplay between urban growth and climate change, emphasizing the need for adaptive urban planning strategies. This study contributes to the understanding of urban growth dynamics in rapidly developing, oil-rich nations with arid climates, offering insights for sustainable urban development and climate resilience in Kuwait and similar contexts.

## 1 Introduction

Today, rapid urbanization represents a global phenomenon characterized by the expansion of existing cities and the development of new urban centers. As cities expand, they transform landscapes and profoundly affect environmental sustainability, economic development, and human well-being. Also, they influence regional and global climate patterns. This rapid urbanization drives uncontrollable population growth, deforestation, depletion of green spaces, and pollution [1–4]. This is why rapid urbanization poses significant challenges for adaptation to climate change, particularly in regions experiencing fast-paced development. This intricate relationship between urban growth and climate change presents both challenges and opportunities for sustainable development, particularly in regions experiencing rapid urbanization. In the context of the Middle East, and specifically in Kuwait, these dynamics take on unique characteristics. Kuwait, a key economy in the Arabian Peninsula, has experienced rapid urban transformation in recent decades, primarily along its coastline. Driven by oil wealth and ambitious development plans, Kuwait had seen its urban areas expand rapidly, with over 98% of its population now residing in urban centers after the year 1984 and until the year 1996 when this percentage increased [5]. This rapid urbanization, coupled with Kuwait’s arid climate and coastal location, makes it particularly vulnerable to the impacts of climate change, including rising temperatures, water scarcity, and potential sea-level rise [6]. Understanding the patterns, drivers, and potential future trajectories of urban growth in Kuwait is crucial for informed urban planning and climate adaptation strategies. In that context, this study aims to forecast and characterize urban growth, and to cross-analyze urban growth with anticipated temperature and precipitation information in Kuwait. By integrating land use/land cover (LULC) data with climate projections, this research offers a comprehensive assessment of urban expansion dynamics and their vulnerabilities to climate change. The analysis and forecasting of spatiotemporal LULC dynamics fulfill several objectives, including (a) revealing the fundamental mechanisms that underlie these dynamics [7], (b) predicting future LULC transitions [8], and (c) supporting sustainable planning for the management of natural resources, infrastructure, and urban areas [3]. Additionally, examining both actual and projected built-up patterns is advantageous for (a) avoiding or reducing the impacts of negative environmental risks and (b) conserving natural resources [9–11]. In this context, the main parameter employed is the geographic scope, precisely the spatial extents of cities. The compact city model, first presented by Dantzig and Saaty in 1974, promotes the amplified density in urban areas [12]. This prototype represents the theoretical foundation for the current study and guides the analytical exploration of urban form and its implications for environmental sustainability. However, to foster cities with enhanced and considered sustainability, it is essential to develop a more comprehensive understanding of city morphology that extends beyond mere density. Urban morphology is naturally associated with many growing aspects, including prevailing technological, political, cultural, social, and economic conditions. These aspects are not inert; they continuously evolve due to multiple influences, as demonstrated by investigations carried out by Frankhauser (2015), McAdams (2007) and Batty and Xie (1996) [13–15].

The research question of this paper focuses on investigating and monitoring (i) the dynamic temporal changes in the effect size of key urban development drivers, and (ii) the utility of these dynamics for the proactive development of new master plans that must be adapted to upcoming changes in urban configurations due to various factors, such as climate change. Moreover, the methodological approach developed in this paper can potentially be applied to other rapidly urbanizing regions, particularly in arid and semi-arid environments facing similar challenges. By bridging the gap between urban growth modeling and climate change vulnerability assessment, this study contributes to the growing body of literature on sustainable urban development in the face of global environmental change. It also provides valuable insights for policymakers and urban planners in Kuwait and beyond, as they grapple with the challenges of managing urban growth while building resilience to climate change impacts.

### 1.1 Scientific lacuna and research question

The present research aims to investigate and monitor (i) the dynamic temporal changes in the effect size of the key urban development drivers, and (ii) the utility of using these dynamics for the proactive development of new master plans that must be adapted according to upcoming changes in urban configurations, due to different factors as the climate change, for instance. Such master plans are critical for infrastructure development, resource allocation, sustainable growth, and social equity.

However, to the depth of the authors’ knowledge, limited research has been carried out to date for exploring and analyzing (i) the aforementioned dynamic temporal changes in the effect size of driving factors and morphology of urban developments within the Kuwait territory and (ii) their vulnerability against the climate change (mainly the changes in the spatial distribution of temperature).

### 1.2 Urban morphology and sustainable development

Urban sprawl has long been identified as a major impediment to sustainable development. This urban expansion is intensifying at the expense of natural lands, which encourages the residents in peripheral areas to depend more on car traffic, fossil fuel use, and thus emitting more pollutants. Notably, the intensive construction of roadways has contributed to increased pollution, higher greenhouse gas emissions (GHG), and greater consumption of natural land compared to external metropolitan areas.

Politicians, the media, and the scientific community have typically advocated for vertical, dense, and compact cities as the solution. They argue that this model promotes public transit usage, better access to amenities and services, and enhanced social connectedness [12]. Nevertheless, the effectiveness of compact city strategies has been called into question by other authors [16,17], who have shown that these policies can lead to (i) reduced access to green and natural spaces, (ii) increased road congestion, and (iii) higher estate prices. Given these potential drawbacks, a qualitative analysis of the patterns of new urban expanses is an important sustainability matter. This research paper adopts a comprehensive approach to predicting and understanding the spatial patterns of urban development, as well as the extent of the influence of key urban drivers. The prediction process is based on the artificial neural network of the Multilayer Perceptron Markov Chain Modelling (MLPMCM) approach. The MLPMCM, a type of artificial neural network, is well-suited for predictive modeling of complex spatial associations and their driving factors.

This structural breakdown improves the strength of the spatial analysis, providing a detailed comprehension of the spatial mechanism and encouraging informed practice and regulating procedures. Subsequently, a description of the corresponding technical context is provided.

### 1.3 Scope, originality, and outline of the paper

This research forecasts future LULC configurations for 2050 and 2100, with its originality lying in the assessment and analysis of the dynamics of key driving factors over time. This research employs existing and actual LULC data maps for the years 1985, 2005, and 2022, within the application of the MLPMCM model, which, in turn, is used to forecast future LULC distribution. The simulation model integrates numerous data referring to the main drivers of urban development, to predict forthcoming LULC configurations in the horizon of years 2050 and 2100. The incorporation of these factors as well as assessing the accuracy and skill measure of different combinations of these factors enables the evaluation of the extent of impact applied by these driving factors on urban expansion over different temporal periods. In other words, by incorporating a temporal dimension into the MLPMCM, this research aims to provide a more nuanced understanding of the dynamic relationships between drivers and LULC outcomes in Kuwait. The study employs time-varying coefficients of accuracy and skill measure within the MLPMCM framework to capture the evolving influence of drivers on LULC patterns. This approach allows the analysis of how the effect sizes of key factors fluctuate across different periods, reflecting, to some extent, the changing socio-economic, environmental, and policy contexts. For instance, the impact of economic growth on urban expansion might be more pronounced in the early stages of development but diminish over time as the focus shifts toward sustainable urban planning. Similarly, the influence of environmental regulations on agricultural land use could intensify as climate change concerns become more pressing. By analyzing these temporal dynamics, the research uncovers the underlying mechanisms that shape LULC changes in Kuwait and how they adapt to shifting circumstances. Thus, in more detail, the originality of this study lies in its ability to capture the evolving nature of driver-LULC relationships, which is often overlooked in traditional LULC modeling approaches. By incorporating time-series analysis techniques into the MLPMCM framework, the research provides a comprehensive and realistic representation of the complex interplay between socio-economic, environmental, and policy drivers in shaping Kuwait’s land use landscape. The findings of this study are expected to have significant implications, for policymakers and urban planners in Kuwait, in justifying the implementation of infrastructure and energy-related initiatives, particularly within the context of sustainable development. By visualizing the changing effect sizes of drivers over time, interactive dashboards could be developed to enable stakeholders to anticipate future LULC changes and proactively develop strategies to address emerging challenges. This innovative approach to assessing the temporal dynamics of driver-LULC relationships represents a significant contribution to the literature, advancing the field of LULC modeling and informing more effective land use planning in Kuwait and similar contexts. Regarding future urban growth, the research hypothesis posits that human activities, including demographic growth and economic development, are the primary drivers. Moreover, the vulnerability of the predicted future urban growth is preliminarily assessed using spatial analytics in relation to predicted climate change. While this research provides valuable insights, it acknowledges certain limitations. Due to constraints related to data accessibility and availability, influential urban determinants such as political and socio-economic variables, including land use policies, accessibility, employment density, and income levels, have not been incorporated into the analysis.

The exclusion of these factors may limit the comprehensive understanding of the complex interplay between various drivers of urban growth and development. A further constraint is that the used MLPMCM did not account for the evolving nature of infrastructure development across the studied time periods. This means that the predictions, mainly founded on present data, may not supply a fully precise representation of forthcoming trends, as they do not capture the evolving nature of these factors [18–21]. Moreover, it should be noted that (a) assessments of climate change vulnerability may be influenced by uncertainties inherent in climate prediction models, and that (b) the findings and conclusions drawn from this research may have a degree of regional specificity, being most directly relevant to the Kuwaiti context. Nonetheless, the core validity of the research findings remains intact, as the study still provides valuable insights within its defined scope. In the following subsections, two crucial aspects of this study are presented: first, the technical underpinnings of the research are presented, establishing the methodological framework; second, the vulnerability and general adaptive capacity of urban development to climate change are examined.

### 1.4 Technical context: Review of multilayer perceptron spatial application in recent literature

Diverse land use/land cover (LULC) forecasting models have been created to analyze earlier spatial changes and simulate future dynamics of the urban pattern. Out of these models, the Multi-Layer Perceptron (MLP) has appeared as a highly operative method in the field of urban development research. The MLP, a type of artificial neural network (ANN), has become a popular choice for modeling various urbanization scenarios. Its ability to capture multifaceted nonlinear patterns makes it well-suited for analyzing land use transitions and simulating urban expansions. The use of the MLP in urban modeling offers distinct advantages. As an artificial neural network, MLP excels at uncovering intricate nonlinear relationships within complex urban datasets and identifying patterns that may not be directly apparent through traditional analytical methods. Moreover, the MLP demonstrates exceptional proficiency in processing large datasets containing numerous variables, making it particularly well-suited for urban modeling endeavors [22,23]. The MLP neural network has gained recognition for its utilization in modeling LULC transitions. This is primarily due to its use of training rules known as “back-propagation”, which serves as the foundation for the MLP’s learning process. Back-propagation is a process that fine-tunes the weightiness of the network’s connections based on gradients and errors. The model of MLP comprises at least an input layer, where data is fed into the network, one or more hidden layers that process the information, in addition to one or more output layers that generate the final predictions, where each neuron in a layer is associated to neurons in adjacent layers [24]. To improve the predictive competencies of future spatiotemporal dynamics of urban growth and LULC, the MLP is often coupled with the Markov Chain Model (MCM) [25–27]. The MCM provides a probabilistic framework for understanding the historical transitions between different LULC classes, while the MLP captures the complex spatial relationships and nonlinear patterns within the data [28,29]. The MLP-MCM model has been widely used to detect diverse drivers affecting the evolution of the urban fabric. The literature review on urban evolution models, directed by Kim et al. (2020) [30], highlighted a variety of natural, infrastructural, and socioeconomic drivers that steer land use dynamics. Building on this, many analyses have implemented the MLP-MCM to project future LULC patterns.

For instance, Kamaraj and Rangarajan (2022) [31] employed the MLP model to forecast LULC dynamics in the Bhavani basin, India. In their study, the researchers used the spatial distribution of five driving factors, including proximity to pre-existing urbanized regions, proximity to roadways, topographic aspects, slope, and elevation to assess their potential impacts on LULC dynamics. The results of this research revealed a precise MLP projection of future LULC dynamics within the Bhavani basin in India from 2025 to 2030. The analysis stated that proximity to road networks, proximity to urbanized areas, and topographic elevation are anticipated to have substantial influences on future urban growth. In a similar vein, Ozturk (2015) [23] combined the Cellular Automata model with the MLP Markov Chain Monte Carlo model to forecast urban expansion in the Atakum district in Samsun, Turkey. This modeling structure unified a range of spatial parameters, including proximity to coastal boundaries, river features, railway lines, primary road networks, and urban centers in addition to topographic slopes. Particularly, the MLPMCM model has been extensively utilized in various scientific studies to forecast and analyze future urban dynamics throughout different scenarios. These include investigations by Yonaba et al. (2021) [32] in Burkina Faso, Benavidez-Silva et al. (2021) in Chile [33], Armenteras et al. (2019) in the Colombian Amazon [34], Mirici et al. (2017) in Turkey [35], Liu et al. (2017) in China [36], Hasan et al. (2017) in Bangladesh [37], Wang et al. (2016) in Connecticut in the United States [38], and Mishra and Rai (2016) in India [39]. Nevertheless, to the best of the authors’ knowledge, previous research in this field has been limited in its investigation of the implications of spatial parameters and causal elements, as well as the extent of their influence on urban dynamics.

### 1.5 Climate change resilience in urban environments

Urbanized zones are prone to the effects of climate change because of different characteristics like dense demographic agglomeration and vulnerability to severe weather conditions [40]. Emerging research indicates that the rapid expansion of cities is propelling environmental transformations at a historically unparalleled first-time magnitude, and is a significant driver of Greenhouse Gas emissions (GHG) [41–43]. Climate change features, such as rising surface and air temperatures, more severe weather conditions, and changing rainfall patterns, significantly impact urban areas [44]. These impacts affect economies, urban infrastructure, and public well-being [40,45,46]). One of the prime influences of climate change for urbanized areas is the rise of temperatures, which is commonly linked to the phenomena of Urban Heat Islands (UHI) [4]. This UHI effect can intensify heat-related health issues, drive-up energy consumption for cooling buildings, overburden the electrical grid, increase demand for drinking water, elevate the danger of urban and wildfires, and potentially compromise the integrity of infrastructure [45]. Additionally, climate change encourages alterations in precipitation patterns. Intensified rainfall patterns resulting from increased precipitation events can cause flooding, disrupting transport, harming vegetation and infrastructure, and forcing the public to relocate. Equally, rare rainfall in other areas may induce droughts. These variations in precipitation patterns could affect both urban and rural areas, influencing their water resources, agronomic practices, and drainage networks [47–50]. The susceptibility of urban areas to climate change differs based on various aspects, such as social characteristics, economic state, infrastructure, demographic density, and geographic position. For example, littoral conurbations face greater risks of rising sea levels and flooding, whereas inland-located cities might be more prone to drought conditions [45].

Cities with high population density are particularly susceptible to thermal stress and heat-related health issues. Additionally, urban areas with old infrastructure are at greater risk of suffering damage from severe weather phenomena like flooding [45]. Cities that have struggling economies and significant social inequities are more susceptible to the effects of climate change and have lower adaptation abilities [51]. Furthermore, the weakness to adapt to climate change is especially seen in cities, where accelerated urban expansion and densification can intensify the influences. More specifically, the course of urban expansion can intensify climate change risk by modifying local environments and intensifying the vulnerability to these risks. As an example, Erlwein et al. (2023) emphasize the competing priorities of providing housing and maintaining green spaces in cities, which can make it challenging for policymakers to navigate the complexities of urban densification and climate change adaptation [47]. Similarly, increased urban densification can result in a decrease in green spaces, which increases cities’ adaptiveness to climate change effects such as thermal stress [50]. Additionally, built-up development can place significant pressure on water sources, making adaptive managing plans essential for sustainable usage. Furthermore, changes in the patterns of the natural water cycle can raise the risk of both floods and droughts, as these trends are estimated to become more extreme and prevalent as a result of climate change [49]. Various dimensions of climate change effects such as social, environmental, physical, and economic impacts can affect the form of urban development, particularly in areas experiencing significant urbanization. For instance, areas with increased physical vulnerability might require the adoption of tailored codes for buildings or LULC regulations to mitigate risk. More, climate change can impact how services and resources are allocated in expanding built-up spatial extents, possibly resulting in social disparities. Similarly, the vulnerability can also (a) impact investment trends, thereby diverting the economic growth of emerging built-up areas, and (b) inform the integration of green and blue infrastructure and environmentally-based adaptation plans into urban master planning [48]. Nevertheless, a range of additional adjustment and mitigation strategies can be formulated. This involves improving current masterplans to reduce the effects of surface UHIs, for instance, by incorporating water parks and green spaces in city centers and their suburban areas. Additionally, the mitigation measures comprise investing in green infrastructure like rain gardens and rain trees to effectively handle rainfall runoff and enhance underground recharge and water infiltration. Other measures may involve building water channels and seawalls, along with various protective strategies to prevent flooding. Enhancing water supervision systems to address floods and droughts, implementing initial warning schemes for severe weather conditions, and raising public awareness about the risks of climate change and the corresponding mitigation policies have also been put first [40]. The relationship between climate change and urban expansion is complex and reciprocal, making it essential to incorporate climate vulnerability assessments and adaptive measures into urban development plans from their inception. This interplay demands thoughtful analysis to be addressed effectively. Implementing these preventive measures can steer urban growth towards minimizing climate-related risks while enhancing resilience. The following section, titled “Methodology,” includes the selection of the zone of interest as well as a detailed report of the research method, the necessary data, and the techniques used for data collection. Section 3 displays the simulation results of the MLPMCM.

Section 4 analyzes, discusses, and interprets the findings, along with an assessment of climate change vulnerability in the broader context of sustainable urban development and climate resilience in Kuwait and similar regions. Finally, Section 5 recapitulates the findings and proposes new contexts for further research.

## 2 Methodology

This section provides an overview of the selected study area, the formulated research methodology, and the employed data collection techniques.

### 2.1 Study area: Kuwait

Kuwait, geographically positioned at the northeastern corner of the Arabian Peninsula, represents the study area of this research due to its rapid urbanization and distinctive geographical features. Covering approximately 17,818 km², it is bordered by the expansive waters of the Arabian Gulf and neighbored by Iraq and Saudi Arabia. Its urban centers, home to over 4.3 million people, sprawl amidst vast desert and coastal stretches, reflecting a complex urban tapestry [52]. Kuwait City, the capital and largest urban area is characterized by its unique urban form shaped by its coastal location and historical development patterns, offering a rich case study for urban growth modeling. Kuwait has experienced a significant urban shift over the past few decades. In 1975, 89.4% of the country’s population resided in urban areas, a figure that highlighted Kuwait’s early commitment to urban development [5]. By 2020, this percentage was projected to reach 100%, indicating that Kuwait had become fully urbanized [5]. This urban growth, largely propelled by Kuwait’s oil wealth, has not only reshaped its landscape but has also induced significant land use changes, providing a unique lens to examine the interplay between economic growth and urban development [53]. Additionally, Kuwait’s harsh climate, characterized by extreme heat and scant rainfall, amplifies its vulnerability to climate change, thereby magnifying the challenges of water scarcity and heat stress. These climatic factors are critical in assessing the sustainability and resilience of Kuwait’s urban infrastructure [6]. Central to this narrative is the Kuwait National Development Plan, or Kuwait Vision 2035. This strategic initiative aims to further modernize the nation’s infrastructure, diversify the economy, and enhance the quality of urban life. It is an ambitious governmental response to the multifaceted pressures of economic diversification, and environmental sustainability [54]. By focusing on Kuwait, this study aims to contribute to the understanding of urban growth dynamics in rapidly developing, oil-rich nations with arid climates. The findings from this research have the potential to inform urban planning and climate adaptation strategies not only in Kuwait but also in similar contexts across the Middle East and beyond.

#### 2.1.1 The transformation of the Kuwait area.

The spatial reconfiguration of Kuwait’s urban landscape has been marked by significant changes in recent decades. This has included the rapid expansion of urban areas into the surrounding desert, as well as the creation of new commercial districts outside the traditional city center (outside the wall) [55]. Since the 1950s, Kuwait’s government has designed urban planning master plans aimed at promoting concentrated growth in the central business district (CBD) and establishing low-density residential zones in the areas surrounding it [56]. The urban transformation was characterized by a shift from the historic, densely populated areas near the coast to sprawling suburban developments, often following a car-centric model [57]. One of the ambitious urban development plans in Kuwait has been the initiative to construct 12 new cities, driven by the need to accommodate a growing population, diversify the economy, and enhance the overall quality of life for residents [58]. Moreover, a key element of these urban expansion efforts has been revealed as land reclamation, which has played a pivotal role in addressing Kuwait’s limited land area. In this context, major projects like the Kuwait City waterfront expansion and the ambitious Sabah Al-Ahmad Sea City have not only extended Kuwait’s coastline but also significantly reshaped its urban landscape. Unlike most recent Gulf coastal developments, which typically infill valuable inshore marine habitats, Sabah Al-Ahmad Sea City was designed to be built on a low-lying coastal desert in southern Kuwait. This innovative approach brought the sea to the desert, creating a new urban area that will eventually house around 100,000 people, without disrupting existing marine ecosystems [59]. These developments have expanded the available land for urban growth while, simultaneously, redefining the spatial and functional layout of Kuwait’s urban areas. In tandem with these large-scale urban expansion efforts, the Kuwaiti government has played a crucial role in shaping the urban landscape through policies encouraging citizens to settle in expanded urban cores within established suburban areas. These policies have also facilitated the redevelopment of the old city, leading to the emergence of modern residential neighborhoods and reshaping the traditional urban forms of Kuwait’s city center [55]. Together, these initiatives demonstrate the Kuwaiti government’s commitment to modernizing its urban infrastructure, while balancing the need for sustainable development and the preservation of cultural heritage. This urban growth has been further fueled by Kuwait’s oil wealth, which has fundamentally altered the country’s economic landscape. The influx of oil revenues has enabled significant investments in infrastructure and public services, accelerating the modernization of Kuwait’s urban areas [57]. However, this rapid expansion has also brought challenges such as traffic congestion, environmental degradation, and social stratification, raising concerns about sustainable development and equitable urban growth [57].

#### 2.1.2 Detailed urban dynamics in Kuwait.

Kuwait has experienced a dramatic population surge since the latter half of the 20th century, transforming its urban landscape and intensifying demands on its vital coastal resources. In 1961, shortly after independence, the population stood at a modest 321,621. However, by 2012, this figure had skyrocketed to approximately 3.26 million, according to the Kuwait Government Online Portal [60], representing a tenfold increase in just over five decades. This exponential growth has been primarily concentrated in Kuwait City and its surrounding coastal areas, which have undergone significant spatial and demographic changes. The coastal strip has experienced about 4% annual population growth [61]. This concentration is not merely coincidental but is intrinsically linked to the critical resources this region provides. Kuwait’s coastal zone holds paramount importance as the main source of both fresh water and electricity for the burgeoning population. The narrow coastal strip is home to several desalination plants that form the backbone of Kuwait’s water security, producing the majority of the country’s potable water supply [62]. This is crucial in a country where natural freshwater resources are scarce. Paralleling the water production facilities, Kuwait’s power plants are also strategically located along the coast.

These plants are energy-intensive, largely due to the high demands of the desalination process, and consume significant amounts of fossil fuels. The intertwined nature of water and energy production in Kuwait highlights a critical challenge: as the population grows and urbanization intensifies, so does the demand for both water and electricity, putting increased pressure on these coastal resources [63]. These transformations have been driven by a combination of factors, including the Kuwaiti government’s socio-economic agenda introduced in 1952 to channel oil revenues into development programs [64]. This agenda included a large-scale urban renewal scheme that aimed to modernize the country’s infrastructure and housing [64], especially with the demographic composition of Kuwait which includes an additional layer of involvedness, with a high number of non-citizens. Thus, the population distribution, of citizens and foreigners, coupled with the government’s ambitious plans for new cities, highlights the need to consider the social preferences and needs of both local and international residents in the urban planning process similar to other Gulf Cooperation Council (GCC) countries [65].

#### 2.1.3 Neighborhood typologies.

The diverse neighborhood typologies in the Kuwait metropolitan area reflect a blend of traditional and modern architectural styles that cater to the evolving urban landscape. Traditional Arab-style courtyard houses were replaced with modern buildings, while suburban developments now showcase modern villas and high-rise apartment buildings in down town areas [66]. In the old town center of Al-Asimah Governorate, the traditional urban forms of dense, low-rise buildings and narrow streets preserve the historical architectural style. In contrast, the post-oil era brought about the development of planned suburbs, featuring grid-like street patterns and detached housing. This illustrates a shift toward modernist urban planning [67]. Meanwhile, urban centers such as Kuwait City have expanded vertically with high-rise commercial and residential buildings. Over the years, these urban forms have undergone significant transformations, driven by broader social and economic changes. The trend towards larger residential plots and car-dependent layouts has reshaped the urban landscape, reflecting a preference for spacious living environments that prioritize privacy and individualism, often at the expense of public shared spaces and pedestrian-friendly designs [57]. The expansion into suburban areas has led to an increase in single-family homes, meeting the desires of many Kuwaiti nationals for detached housing and thus contributing to urban sprawl. As a result, the once vibrant, interconnected neighborhoods characterized by walkable streets have been replaced by segregated zones that emphasize automobile use. This evolution reflects the complex interplay between cultural preferences, economic factors driven by oil wealth, and urban expansion initiatives that prioritize pavilion-like houses over sustainable community design [57].

#### 2.1.4 The infrastructure as a catalyst and promoter of urban development.

Kuwait’s urban transformation is being driven by major infrastructure development projects, such as the expansion of Kuwait International Airport, the implementation of the 4th Kuwait Master Plan, and the enhancement of existing port infrastructure. These initiatives, alongside road development projects, are key components of the Kuwait National Development Plan (2020–2025), designed to improve connectivity, guide spatial expansion, and attract further developments [68]. This large-scale infrastructural expansion is essential for meeting the needs of the growing population and promoting economic diversification.

**Fig 1:**
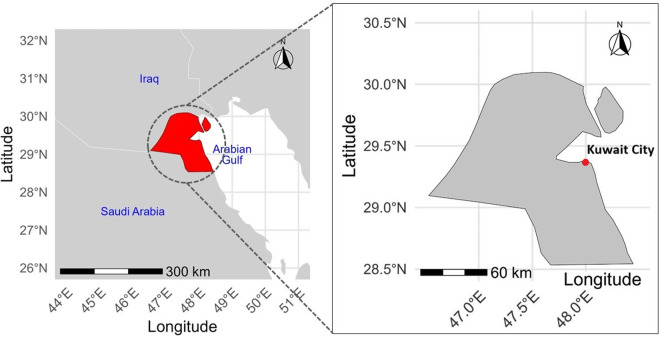
Geographic location of the study area.

To effectively manage this rapid urban growth, it is important to understand the ongoing dynamics of urban form transformation. While the radial sprawl of Kuwait’s metropolitan area plays a central role, regional dynamics within key “corridors” also strongly influence mobility and land use patterns [58]. These dynamics help shape the broader territorial organization of Kuwait, ensuring that infrastructural developments align with the city’s spatial and mobility needs. Fig 1 illustrates the geographic location of the study area.

### 2.2 Methodological framework and data collection

The findings of this research provide insights into the evolution of LULC over time, as well as the influences of these dynamics on urban development and the adaptation methods against climate change. The forecast procedure in this section utilizes the MLP-MCM, accounting for several key driving factors. The concluding segment of the methodology involves evaluating the vulnerability of existing and future urban settings to climate change. This evaluation is conducted by analyzing temporal changes in air temperature, and assessing (through estimation) how these variations may influence urban fabrics, particularly in relation to drought and increases in land surface temperature. Furthermore, a temporal change analysis is performed to identify the evolution of urban patterns and to detect alterations in the spatial distribution of land cover over time. The methodological process employed in this study to predict future spatial patterns of LULC for the years 2050 and 2100 utilizes actual LULC maps of Kuwait for the years 1985, 2005, and 2022. This approach also enables the assessment of urban dynamics and climate change vulnerability for both the actual and anticipated future urban areas of Kuwait. The core of this prediction methodology is the machine learning-based Multi-Layer Perceptron Markov-Chain Model (MLPMCM). The MLPMCM model is incorporated within the “Land Change Modeler” of the TerrSet 18 software. The prediction process involves several key steps: (a) selecting the main LULC transitions of interest, (b) training the MLPMCM model, (c) securing a satisfactory overall accuracy rate and skill measure of the model, (d) checking the validity of the predictions power against the actual base data, and (e) performing the final LULC runs to forecasts for 2050 and 2100 using the fitted transition model.

Thus, the ability of the MLPMCM model to generate robust forecasts of future LULC patterns, as well as to enable the analysis of associated urban dynamics and climate change vulnerability for the Kuwait region, is highlighted as a key strength of this methodology. The choice of driving factors is informed by recommendations from previous studies [69–72]. The following outlines the data needed and the corresponding data collection techniques employed in this study.

#### 2.2.1 Data collection sources.

The residential and commercial mean prices in Kuwait for the year 2015 are extracted from the publication of Mostafa (2018) [73] which are in turn obtained from the 2016 Annual Report of the National Bank of Kuwait [74]. The LULC data are downloaded from the dataset of the first global 30 m land-cover dynamics monitoring product [75,76] using a detailed classification system for the period from 1985 to 2022, developed with the help of the continuous change detection method and dense time-series Landsat imagery. The LULC configuration comprises seven land cover classes: Urban, agriculture, forests, bare soil, natural vegetation, wetland, and water bodies. LULC data layers were sourced with a spatial resolution of 30 meters, which aligns with the classification system applied for Landsat imagery. This resolution standardization allows for consistent spatial analysis across different time points, ensuring accuracy in capturing LULC changes over time. The datasets were aligned to maintain consistent LULC categories (such as urban, agricultural, forest, bare soil, etc.) across the different years. This step is critical in allowing accurate analysis of transitions and maintaining consistency in land cover classification for model training. The 30 meters population density maps of Kuwait for the years 1985, 2005, and 2022 are extracted from the population dataset of the Global Human Settlement Layer (GHSL) population grid multitemporal (1975–2030) prepared by the European Commission’s Joint Research Centre (JRC) [77]. The Digital Terrain Model (DTM), of 30-meter spatial resolution, is extracted from the Ensemble Digital Terrain Model (EDTM) of the world [78]. The Slope map is then generated in ArcGIS pro software based on the DTM data. The data of open street map, obtained from the Geofabrik database [79], is used to generate Euclidean density, line density, and point density maps of diverse driving factors including roadways, parking spots, fuel stations, industrial areas, commercial areas, residential areas, and waterways. More, climate change data to the horizon of the year 2100, particularly the change in the spatial distribution of temperature patterns are extracted from the database of FAO (2024) [80]. These projections, prepared under the “Regional Initiative for the Assessment of Climate Change Impact on Water Resources and Socio-Economic Vulnerability in the Arab Region” (RICCAR), with a 25-kilometer resolution are based on the RCP8.5 scenario which stands for the Representative Concentration Pathway 8.5. The RCP 8.5 represents a high greenhouse gas concentration scenario that considers a future trajectory where greenhouse gas emissions continue to rise significantly throughout the 21st century, leading to an increase in global average temperature. Specifically, RCP 8.5 is characterized by a radiative forcing level of 8.5 watts per square meter by the year 2100, which corresponds to a scenario with high levels of carbon dioxide and other greenhouse gases in the atmosphere. Three regional climate models dynamically downscaled from RCP8.5 projections are employed by this dataset: CNRM-CM5, EC-EARTH, and GFDL-ESM2M. For the analysis in this paper, the outputs of the EC-EARTH model, at a 25-kilometer resolution, were specifically used as the best performance for the Arabian Peninsula region was shown when compared to historical observations.

While a more comprehensive uncertainty assessment would be provided by using multiple models, a focus on EC-EARTH was chosen for two key reasons: 1) its superior performance in regional validation, and 2) the intent to provide a “worst-case scenario” assessment for urban planning purposes, as more pronounced temperature increases tend to be projected by EC-EARTH compared to other models in the ensemble.

#### 2.2.2 Predictive modeling technique.

Furthermore, the MLPMCM model utilizes historical LULC transitions to gain insights into the underlying LULC dynamics. This understanding is then applied to forecast the potential future LULC configurations. In this context, the Evidence Likelihood (EV), which quantifies the probability of a particular LULC transition based on historical data, is employed in the predictive framework. The evidence likelihood values in this study were validated by comparing predicted transitions against actual observed changes between successive time periods (1985–2005 and 2005–2022). The reliability of these likelihoods is demonstrated through the model’s high accuracy rates explained further in the result sections, where evidence likelihood emerged as the most impacting factor with effect size of 33.35% and 33.25% for the 1985–2005 and 2005–2022 periods respectively. The consistency of evidence likelihood’s influence across both time periods supports its reliability as a predictor of LULC transitions. S1,S2 and S3 Figs in appendix 2, show the spatial configurations of the collected data. Based on visual inspection of the spatial distribution, there are some similarities among these factors. For example, the maps of topographic elevation, distance to sea, and distance to commercial and residential areas have higher values in the middle to northern west sides of the study area. This is because urban areas, including mainly residential and commercial agglomerations, are concentrated in the middle eastern area on the sea side. In the same context, higher values of population density for the years 1985, 2005, and 2022 as well as point densities of parking and fuel stations are located in the Middle Eastern area next to the sea side.

**Fig 2:**
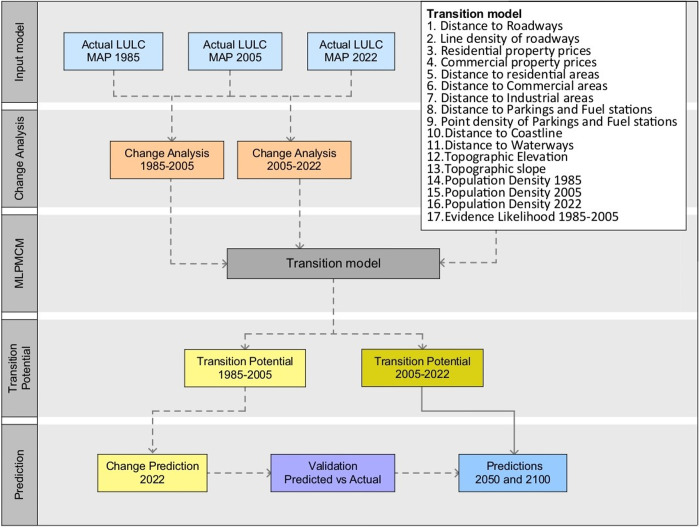
The MLPMCM prediction methodology.

Fig 2 illustrates the MLPMCM prediction methodology.

## 3 Results

The results section presents the findings from the MLP-MCM simulations, as well as the observed and predicted changes in historical and projected urban growth patterns. This comprehensive presentation is divided into three key subsections. These subsections focus on the preselection of transition models in addition to the evaluation of the prediction adequacy of the transition models for the 1985–2005 and 2005–2022 time periods, respectively. This includes an in-depth examination of the specific transitions used in the models, an assessment of the model attributes that capture the skill breakdown and the impacts of the driving factors, and a detailed validation process to test the models’ accuracy.

### 3.1 Shortlisting transition models: Principal LULC transition

**Fig 3:**
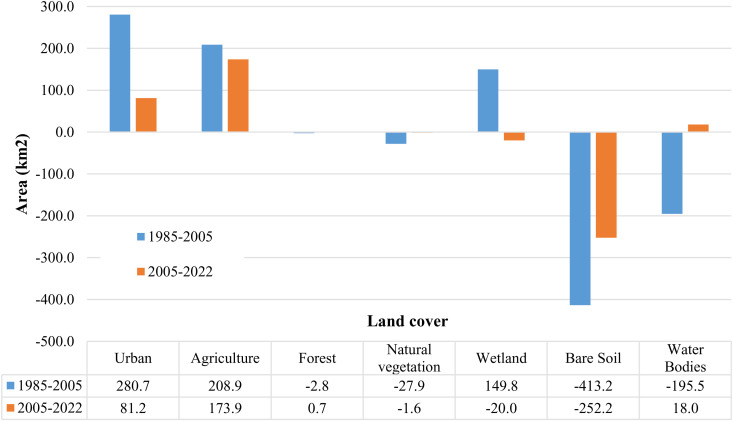
Net changes in LULC areas over the periods 1985-2005 and 2005-2022.

**Fig 4:**
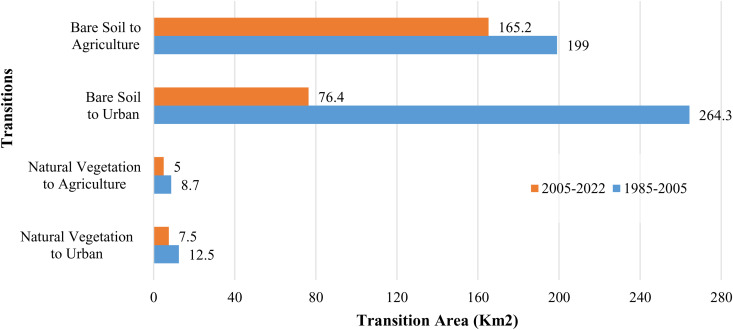
The change in areas, among the 1985-2005 and 2005-2022 periods, of the main transitions (towards urban and agriculture areas).

The net changes in total areas of LULC classes, mainly to urban growth and agricultural areas, are employed to preselect the key transitions to be integrated into the training model. The change values are presented in Fig 3. Significant changes are mainly observed for urban growth, natural vegetation zones, wetlands, bare soil, waterbodies, and lands devoted to agriculture. For instance, while the urban area experienced accelerated growth from 1985 to 2005 (280.7 squared kilometers), which can be driven by several interconnected factors, (including an economic boom that attracted people to cities for job opportunities, a population growth that strained existing infrastructure, and supportive government policies such as infrastructure development), the urbanization slowed significantly, from 2005 to 2022 to 81.2 squared kilometers, likely due to the maturity of urban centers reaching saturation, or the change of urbanization patterns from sprawl to more promoted vertical cities, thereby limiting further urban expansion. Similarly, while agricultural land experienced continued expansion during both 1985–2005 (208.9 squared kilometers) and 2005–2022 periods, driven by several interconnected factors (including rising food demand due to a growing population and changing dietary habits, the trend towards land conversion for crop cultivation, and technological advancements that enhance productivity) growth rates slowed from 2005 to 2022 to 173.9 squared kilometers. This decrease in expansion can be attributed to the trend toward market saturation, which reduces the incentive for further agricultural growth. More, the total area of wetlands increased by 149.8 square kilometers during the period from 1985 to 2005. On the other hand, a significant decrease in the total area of bare soil lands was observed during both studied periods. This decrease, amounting to 413.2 square kilometers in the first period and 252.2 square kilometers in the second, occurred in favor of expanding urbanized and cultivated areas, as well as the growth of wetlands. Furthermore, the total area of water bodies decreased during both periods (1985–2005 and 2005–2022) due to the conversion to various land cover types, particularly bare soil. The change in areas, among the 1985–2005 and 2005–2022 periods, of the main transitions (towards urban and agricultural areas) is presented in Fig 4.

In the second part of the selection process, the transitions considered are those that (i) have high accuracy rates and (ii) contribute to higher values of the overall skill measure for the transition models. More details on the skill measures of the transition models are presented in the next section).

The transitions utilized in the adopted MLP-MCM models are shown in Table 1.

**Table 1:** The MLPMCM transitions model (Periods 1985-2005 and 2005-2022).

1985-2005		
Transition	Transition Accuracy (%)	Area of transition (km²)
Natural Vegetation to Urban	92.3	12.5
Natural Vegetation to Agriculture	90.4	8.7
Bare Soil to Urban	85.6	264.3
Bare Soil to Agriculture	85.5	199
Bare Soil to Wetland	92.7	23.3
Water Body to Bare Soil	84.2	64.8

**2005-2022**		
Transition	Transition Accuracy (%)	Area (km²)
Natural Vegetation to Urban	92.5	7.5
Natural Vegetation to Agriculture	82.4	5
Bare Soil to Urban	84.8	76.4
Bare Soil to Agriculture	84.9	165.2
Bare Soil to Waterbodies	95.82	20
Bare Soil to Natural Vegetation	82.5	10.8

### 3.2 Transition model 1985-2005

The Land Change Modeler component within the TerrSet 18 software was utilized to train the predictive model using a dynamic learning rate and an automatic training process. The model building consisted of 17 input neurons (representing the driving factors), 14 hidden neurons, and 9 output neurons. The automatic training process is employed to enhance the forecast precision of the neural network. This process involves adjusting the neuron weights in the network in order to minimize the error between the predicted outputs and the original data. After 10,000 training iterations, the model achieved an overall accuracy of 78.64% and a skill measure of 0.7597, which represents its ability to forecast future figures. It is worth noting that he model’s accuracy was evaluated by comparing its predictions against actual LULC data as cross-validation accuracy verification. The accuracy values were calculated during the training process of the Multi-Layer Perceptron (MLP) neural network.

#### 3.2.1 Model skill breakdown by transition (1985–2005).

The “model skill”, which represents the model’s ability to predict future data, is generally divided into several sub-model skills that correspond to the different LULC transitions used in the model. This breakdown reveals that “Bare Soil to Wetland” transition has the highest skill of 0.872 followed by 0.809 for “Bare Soil to Urban areas”, 0.769 for “Water Body to Bare Soil”, 0.709 for “Bare Soil to Agriculture”, and 0.708 for “Natural Vegetation to Urban areas”. The least sub-model skill (0.657) is observed with the “Natural Vegetation to Agriculture” transition.

The attributes and the impact values of driving factors of the overall 1985–2005 transition model are presented in S1 and S2 Tables of appendix 1. For the overall transition, the evidence likelihood 1985–2005 is the most impacting factor, with an effect size of 33.35% and a skill measure of 0.25. The subsequent three effective drivers are the distance to roadways, the distance to the coastline, and the distance to commercial and retail areas, with accuracy rates of 12.92%, 12.43%, and 10.29% respectively. The remaining factors (Population Density 2022, Distance to Industrial areas, Elevation, Distance to residential areas, Distance to Waterways, Distance to Parking and Fuel stations, Population Density 2005, Commercial property prices, Point density of parking and Fuel stations, Slope, Line density of roadways, and Population Density 1985) have insignificant influence on the accurateness of the overall transition-model. Subsequently, the hypothesis proposing that anthropogenic-mediated actions, including the spatial distribution of economic activities, population density, and demographic growth, are the primary driving factors of the observed changes is partially supported (particularly by the spatial distribution of road infrastructure and commercial activities). It is worth noting that residential property prices have an extremely lightweight negative impact on the accuracy of the model. This is explained by the inconsistencies of urban growth locations against the spatial distribution of residential property prices. In other words, this is referred mainly to the weak significance and effect of these prices on the location of new urban developments.

#### 3.2.2 The prediction capability of the used multi-layer perceptron Markov-Chain model.

The effectiveness of the MLPMCM model, particularly its forecasting abilities, was thoroughly evaluated. This assessment involved a two-part process: first, the actual LULC data from 1985 and 2005 was employed to predict the 2022 LULC pattern; second, the predicted results were compared to the real 2022 LULC data using TerrSet software. This comparison demonstrated an impressive overall Kappa value (K_Standard_) of 0.95, indicating high accuracy and substantiating the model’s strong predictive capabilities in forecasting future LULC changes [18,81]. A visual representation of the predicted 2022 urban areas is provided in S3 Fig in Appendix 2, further.

### 3.3 Transition model 2005-2022

Forecasting future urban expansion is a key focus of this study. The models examine potential transitions to both urban development and agricultural lands. These transition models use input layers similar to those in the previous model, with one important modification: the evidence likelihood layer now incorporates data from 2005–2022, reflecting more recent historical land use and land cover (LULC) transitions. This update allows the model to capture the most current trends in land use change, providing a more accurate basis for projecting future urban growth and agricultural expansion. The Land Change Modeler component within the TerrSet 18 software was utilized to train the predictive model using a dynamic learning rate and an automatic training process. The model building consisted of 17 input neurons, 14 hidden neurons, and 9 output neurons. The automatic training process is also employed for this transition model to enhance the forecast precision of the neural network. After 10,000 training iterations, the model achieved an overall accuracy of 76.61% and a skill measure of 0.7369, which represents its ability to forecast future figures for the years 2050 and 2100.

#### Model skill breakdown by transition (2005–2022).

The model skill breakdown for the period 2005–2022 is similar to that of the period 1985–2002, in terms of the same transitions and importance order, with some differences in the accuracy values.

This breakdown reveals that “Bare Soil to Waterbodies” transition has the highest skill of 0.855 followed by 0.779 for “Bare Soil to Urban areas”, 0.778 for “Bare Soil to Natural Vegetation”, 0.664 for “Bare Soil to Agriculture”, and 0.657 for “Natural Vegetation to Urban areas”. The least sub-model skill (0.591) is observed with the “Natural Vegetation to Agriculture” transition. The latter low value is justified by the slightest contribution of natural vegetation lands as new areas for agriculture activities. The attributes and the impact values of driving factors of the overall 2005–2022 transition model are presented in S3 and S4 Tables of appendix 1. For the overall transition, the evidence likelihood 2005–2022 is the most impacting factor, with an accuracy rate of 33.25% and a skill measure of 0.25. The subsequent three effective drivers are the distance to the coastline, the distance to roadways, and the distance to commercial and retail areas, with accuracy rates of 13.81%, 11.97%, and 8.48% respectively. The remaining factors (Population Density 2022, Distance to Industrial areas, Elevation, Distance to residential areas, Distance to Waterways, Distance to Parking and Fuel stations, Population Density 2005, Commercial property prices, Point density of parking and Fuel stations, Slope, Line density of roadways, and Population Density 1985) have insignificant influence on the accurateness of the overall transition-model. Subsequently, the hypothesis proposing that anthropogenic-mediated actions, including the spatial distribution of economic activities, population density, and demographic growth, are the primary driving factors of the observed changes is partially supported (particularly by the spatial distribution of road infrastructure and commercial activities). It is worth noting that the residential property prices and the topographic slope have an extremely lightweight negative impact on the accuracy of the model. This is explained by the inconsistencies of urban growth locations with respect to the spatial distribution of residential property prices. In other words, this is referred mainly to the weak significance and effect of these prices on the location of new urban developments.

## 4 Analytical discussion

The initial subsection of this analytical discussion concentrates on illustrating the observed and projected urban dynamics from 2000 to 2050 and subsequently extending to 2100. This comprehensive temporal scope facilitates a holistic understanding of historical urban development patterns alongside future projections generated through the MLP-MCM modeling framework. By encompassing both retrospective analysis and forward-looking simulations, this approach offers a nuanced perspective on the trajectory of urban evolution over an extended timeframe in Kuwait. Building upon this analysis of urban dynamics, the study then proceeds to assess the climate change vulnerability of these projected urban patterns. This assessment will be conducted through a rigorous estimation process, focusing on a single climate scenario and its potential impacts on urban areas in Kuwait. By integrating this specific climate change scenario into the urban growth projections, this analysis aims to provide a focused and detailed understanding of future urban landscapes under particular climatic conditions in the Kuwaiti context.

### 4.1 Urban dynamics over the years 1985 to 2050 and 2100

**Fig 5:**
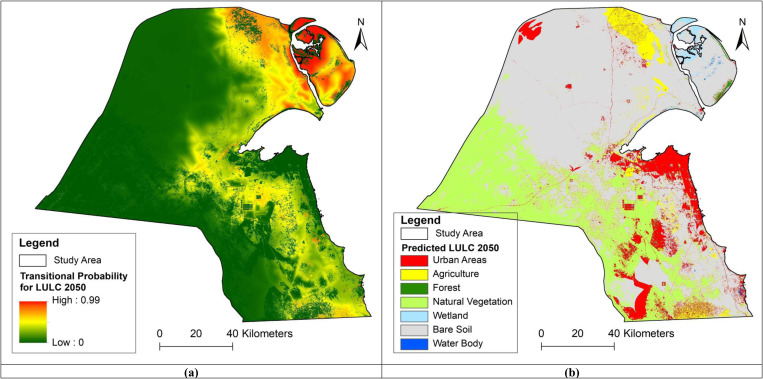
(a) LULC transitional probability map for the year 2050, (b) Projected LULC map for the year 2050.

The predicted expansions of urban areas for the years 2050 and 2100 show that the growth (a) is generally following a space-filling pattern next to the coastal line, and (b) is clustering around the main roads, especially in the North-North-Western and South-South-Western regions. This pattern of growth aligns with Kuwait’s historical development along its coastline and major transportation corridors and highlights the high importance of roadway accessibility and the proximity to the coastline as shown in the model Fig 5.

**Fig 6:**
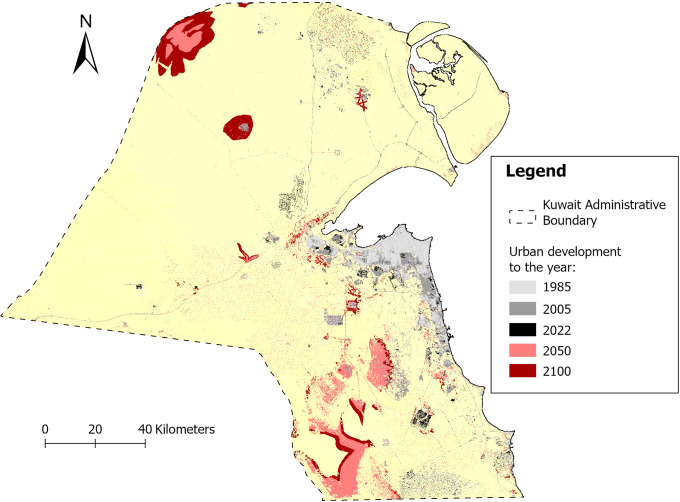
Actual urban areas to the years 1985, 2005, and 2022 and Predicted urban areas for the years 2050 and 2100.

As reported by the results of the prediction simulations, population density seems to have a minor effect on urban growth, with accuracy rates below 2.3% for all periods. This suggests that urban expansion in Kuwait is not primarily driven by population growth or density. It is important to highlight that residential property prices and topographic slope have a significantly minor negative effect on the model’s accuracy. This can be attributed to the inconsistencies between urban growth areas and the spatial distribution of residential property prices in Kuwait. Essentially, this indicates that these prices have limited significance and influence on the placement of new urban developments, likely due to government housing policies as well as the significance of other factors unique to the Kuwaiti context, particularly the proximity to the coastline, road infrastructure, and commercial areas. More, the results of the prediction simulations, the evidence likelihood, representing the historical patterns of land use change, has the highest impact on urban growth in Kuwait, with accuracy rates of 33.35% and 33.25% for the 1985–2005 and 2005–2022 periods respectively. This suggests that past land use patterns are the strongest predictor of future urban development in Kuwait. Following the evidence likelihood, the findings show also the elevated significance of the proximity to the littoral areas as well as the proximity to the road network and commercial/retail areas (8–13% approximately during the two periods 1985–2005 and 2005–2022, respectively) in shaping the spatial form of future urbanization. In the same context, it is worth noting that the importance of the proximity to roadways during the period 1985–2005 was exceeded, during the 2005–2022 period, by that to the coastal line, revealing the importance of the selection of urbanization nearby the littoral line. Nonetheless, slight differences in accuracy rates for the 1985–2005 period within the model are observed for the following factors: (a) proximity to the sea line (12.43%), (b) proximity to roadways (12.92%), and (c) proximity to commercial/retail areas (10.29%). Similarly, slight differences in accuracy rates for the 2005–2022 period within the model are observed for the following factors: (a) proximity to the sea line (13.81%), (b) proximity to roadways (11.97%), and (c) proximity to commercial/retail areas (8.48%). These differences are highlighted by the locations of the forecasted future urbanization, predicted for the years 2050 and 2100. These areas are expected to be located in the North-Northwestern and South-Southwestern regions of Kuwait, linking the country to its neighboring countries, Saudi Arabia and Iraq. Accordingly, these potential locations highlight the importance of proximity to roads and major road networks, as well as main trade and commerce lines. Furthermore, it is anticipated that the future urban structure will support Transit-Oriented Development (TOD) alongside international commerce lines. Thus, the selection of urban growth locations in Kuwait is not primarily driven by population growth, but rather by economic trends and public policy initiatives on urban and economic development. The findings challenge the hypothesis that urban growth is primarily influenced by human behaviors such as population density or migration patterns. Instead, they highlight the significance of economic trends and public policy initiatives in urban and economic development, particularly in the context of Kuwait, where economic factors and infrastructure development play a crucial role. Fig 6 presents previous and forecasted spatial extents of the urban growth in Kuwait over the years 1985, 2005, 2022, 2050, and 2100.

Maps showing the actual and forecasted LULC maps for the years 1985, 2005, and 2022 are presented in S4 Fig in appendix 2.

In terms of change values, the total area of urban zones expanded from 457, 738, and 819 square kilometers in the years 1985, 2005, and 2022, respectively. It is projected to further increase to 1,455 and 1,893 square kilometers by the years 2050 and 2100. The past and forecasted trend of urban evolution seems to follow a quasi-linear trend with an increase of approximately 12 km² per year. This linear growth trend suggests a steady expansion of urban areas in Kuwait, which could have significant implications for land use planning, infrastructure development, and environmental management. The consistent growth rate indicates a need for proactive urban planning to ensure sustainable development and mitigate potential negative impacts on the environment and quality of life. Agricultural land also demonstrates significant growth. From about 94 km² in 1985, it expanded substantially to around 303 km² by 2005. This trend continued, with the agricultural area growing to roughly 477 km² in 2022. Future projections suggest further expansion to about 705 km² by 2050 and a considerable increase to approximately 897 km² by 2100. In contrast, Forest cover shows a stable trend with a total area of about 30 km² in 1985, 2005, and 2022. The projections indicate an unchangingness of forest area by the years 2050 and 2100. For wetlands, the total area was about 170 km² in 2005. The area decreased to roughly 132 km² by 2022. Interestingly, projections suggest a slight increase in wetland area to about 206 km² by 2050, with a further increase to approximately 255 km² by 2100. Natural vegetation shows a relatively stable trend with a slight decline over time. Starting at about 4,635 km² in 1985, it remains nearly constant until 2022, then shows a minor decrease to approximately 4,550 km² by 2050, and further declines to about 4,470 km² by 2100.

Bare Soil, on the other hand, exhibits a significant downward trend. It starts at around 11,900 km² in 1985, decreases to about 11,500 km² by 2005, continues to decline to approximately 11,200 km² in 2022, and is projected to decrease further to about 10,400 km² by 2050 and 9,800 km² by 2100. This paints a picture of significant land use change, with human-managed lands (urban and agriculture) expanding at the expense of bare soil and, to a lesser extent, natural vegetation and forests. The reduction in bare soil could have positive implications for reducing erosion and dust storms. Nonetheless, the conversion to urban and agricultural land may lead to other environmental challenges such as increased water demand, pollution, and habitat fragmentation. Moreover, the projected increase in wetland areas from 2022 onwards is notable and may warrant further investigation into potential causes such as conservation efforts or climate change impacts. The data underscores the need for careful land management and conservation strategies to balance human needs with environmental sustainability to the horizon of the year 2100.

**Fig 7:**
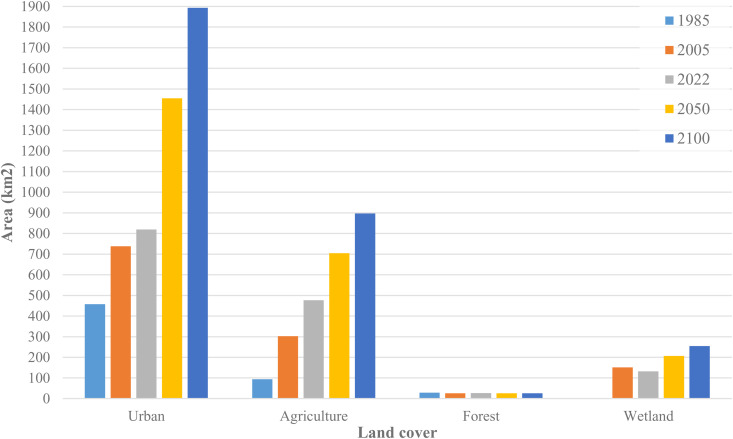
Previous and forecasted tendencies of LULC dynamics.

**Fig 8:**
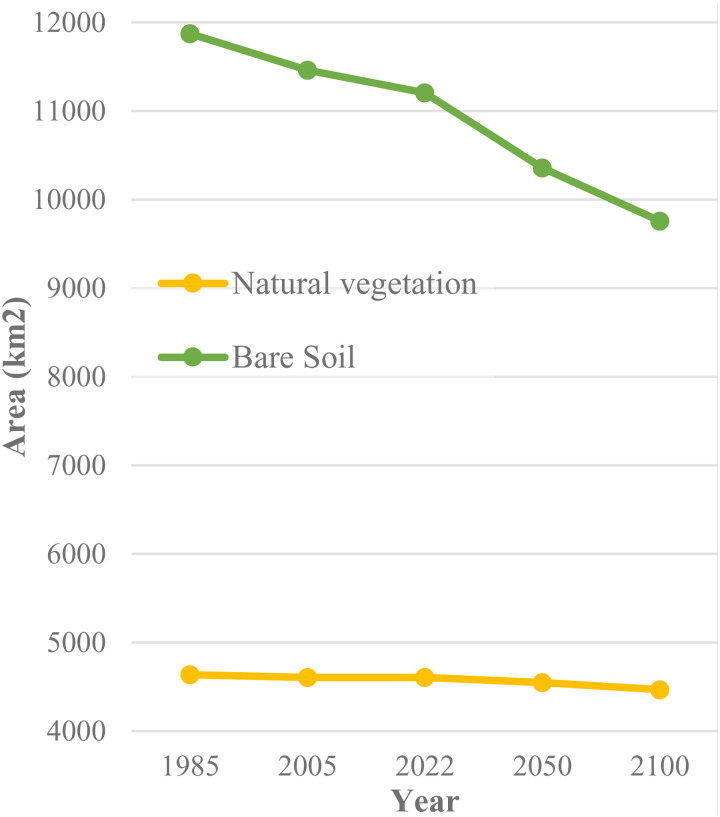
Previous and forecasted tendencies of natural vegetation and bare soil.

Figs 7 and 8 illustrate the previous and forecasted trend of land cover dynamics.

The following subsection discusses the vulnerability of actual and future urban and agricultural areas to climate change.

### 4.2 Vulnerability to climate change

**Fig 9:**
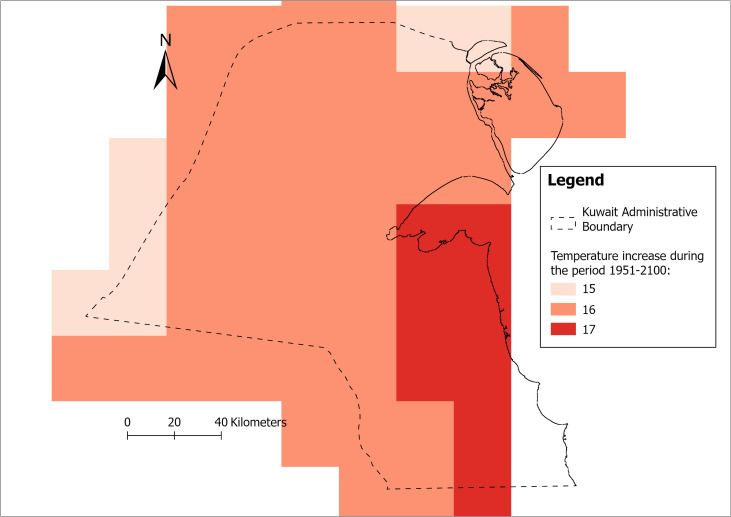
Anticipated climate change 1951-2100: Anticipated temperature increase (data from: ESCWA et al. 2017 [44]).

The model predicts significant urban growth, particularly in the North-North-Western and South-South-Western areas of Kuwait. These areas of expansion need to be planned with climate resilience in mind, considering mainly the projected temperature changes. The analysis of the vulnerability of LULC to climate change, in Kuwait, focuses mainly on temperature and precipitation changes. Based on the RCP 8.5 scenario data from RICCAR [44,80], significant changes in temperature and precipitation patterns are anticipated for Kuwait by 2100. The forecast shows that Kuwait is likely to experience substantial increases in temperature across its territory. The estimated results are subsequently converted into a raster format to enhance visual analysis. The urban and coastal regions are expected to see the most significant temperature rises, with increases of up to 17°C projected by 2100 compared to the year 1951. The temperature change is likely to be slightly lower (1 and 2 degrees Celsius) in the northern and western regions. This warming trend is likely due to a combination of factors, including global climate change, urban heat island effects in densely populated areas, and the country’s arid climate. In detail, urban agglomerations contribute to increased greenhouse gas emissions and obstruct the natural flow of cool air from the Arab Gulf Sea, resulting in higher temperatures. Fig 9 illustrates the changes in temperature over the years 1951–2100. Regarding the changes in precipitation patterns, no definitive climate projections were presented. As urban areas continue to experience changes in climate patterns, it is essential to consider several following implications. For instance, with respect to heat stress, the significant temperature increases, particularly in central and southern coastal regions, pose risks for urban areas in terms of (a) heat-related health issues (as more frequent and intense heat waves, increasing the risk of heat-related illnesses) and (b) increased energy demand for cooling. Additionally, as temperatures rise, the likelihood of droughts and wildfires may also increase. In addition, and with much of Kuwait’s urban development concentrated along the coast, sea-level rise associated with climate change could pose risks to these areas. This may necessitate adaptive measures in urban planning and infrastructure development.

Moreover, the challenging climate conditions may make it difficult to maintain urban green spaces, which are crucial for mitigating urban heat island effects. Innovative urban greening strategies adapted to arid climates may be necessary. The combination of rapid urban growth and projected climate changes presents significant challenges for Kuwait’s urban areas. Integration of these projections into urban planning and development strategies will be crucial for identifying the most vulnerable areas and ensuring the resilience and sustainability of Kuwait’s cities in the face of these environmental changes. The LULC effects of climate change, particularly the temperature changes, can be explored by (a) identifying areas of important LULC change, (b) assessing the current types of LULC as well as those forecasted for the future, and (c) evaluating their resilience to negative impacts as well as their ability to adapt and capitalize on positive opportunities. According to the urban morphology analysis, the fastest growth is expected to take place, as expansions, in the eastern and southern coastal regions of the country, along with certain new areas in the north-north-west and south-south-west sides of the country. This anticipated expansion is driven by various factors, including economic development and population increase. Furthermore, it is anticipated that agricultural lands will expand in the northern-north-eastern and southern-south-eastern regions of Kuwait. The previously presented Fig 5b in addition to Fig 9 illustrate, respectively, the maps of (a) projected urban and agricultural expansions, and (b) areas situated in high climate change (temperature) zones, which could impact thermal comfort in urban areas and cultivation activities on agriculture lands. The relationship between the effects of elevated temperatures and the LULC distribution investigates, specifically, the implications of these changes across various regions. These implications include the increased risk of wildfires, droughts, increased health risks, loss of biodiversity, and the occurrence of warmer and more extreme weather events. Concerning urban growth, the Southern and Eastern coastal regions, as well as the North-North-Western region, are expected to experience high levels of warmer and more extreme weather events. This could lead to high health risks across all these urban growth areas. However, they are projected to have low vulnerability to droughts and wildfires, and a low risk of biodiversity loss. The South-South-Western region stands out among urban growth areas. While it shares the high risk of warmer and more extreme weather events and increased health risks, it shows moderate vulnerability to droughts and wildfires. Additionally, this region is expected to face moderate to high loss of biodiversity, indicating a more complex set of climate change challenges compared to other urban growth areas.

Regarding the expansion of agriculture lands, both the North-North-Eastern and South-South-Eastern regions display high vulnerability across all categories of climate change impacts. These areas are expected to experience high levels of warmer and more extreme weather events, increased health risks, droughts, and wildfires. They also face a moderate risk of biodiversity loss, which is higher than most urban growth areas but lower than the South-South-Western urban region. The consistent “High” ratings across multiple climate change impacts for agricultural expansion areas suggest these regions may be particularly vulnerable to the effects of climate change. This contrasts with the urban growth areas, which generally show more varied levels of vulnerability across different climate change impacts. It is worth noting that all regions, regardless of whether they are designated for urban growth or agricultural expansion, are expected to face high risks from warmer and more extreme weather events and increased health risks. This suggests that adaptive strategies for both urban development and agricultural practices will be crucial across all regions to mitigate these widespread climate change impacts.

Table 2 summarizes the observed vulnerability to climate change.

**Table 2:** Vulnerability of urban growth and agriculture expansion areas to climate change.

Climate change LULC and location		Changes in temperature				
		Warmer and more extreme weather events	Loss of biodiversity	Increased health risks	Droughts	Wildfires
Urban growth	Southern coastal region	High	Low	High	Low	Low
	Eastern coastal region	High	Low	High	Low	Low
	North-North-Western region	High	Low	High	Low	Low
	South-South-Western region	High	Moderate to High	High	Moderate	Moderate
Expansion of agriculture lands	North-North-Eastern region	High	Moderate	High	High	High
	South-South-Eastern region	High	Moderate	High	High	High

The interplay between climate change and urban expansion presents a significant challenge for Kuwait. The country must devise strategies to lessen the adverse effects while also capitalizing on the potential benefits of climate change, particularly in areas such as resources, energy, and productivity. To address these vulnerabilities, Kuwait may need to consider various adaptation strategies as (but not limited to): (a) implementing heat-resistant urban design and building standards, (b) enhancing water management systems, including improved water recycling and efficient irrigation methods, (c) developing climate-resilient infrastructure, particularly in coastal areas, (d) increasing urban green spaces with drought-resistant vegetation to help mitigate urban heat island effects, (e) investing in renewable energy sources to meet increased cooling demands sustainably, and (f) improving public health systems to deal with thermal discomfort and heat-related health issues.

According to the urban morphology analysis, the fastest growth is expected to take place, as expansions, in the eastern and southern coastal regions of the country, along with certain new areas in the north-north-west and south-south-west sides of the country. This anticipated expansion is driven by various factors, including economic development and population increase. Furthermore, it is anticipated that agricultural lands will expand in the northern-north-eastern and southern-south-eastern regions of Kuwait.

## 5 Conclusion and future research

This comprehensive study on urban dynamics and climate vulnerability in Kuwait yields several significant findings and implications for urban planning and climate adaptation strategies. The research, employing the Multi-Layer Perceptron Markov Chain Model (MLPMCM), has forecast land use and land cover (LULC) changes in Kuwait up to the years 2050 and 2100, providing a long-term perspective on urban growth patterns and their potential impacts. The MLP-MCM model, having high accuracy rates, reveals that Kuwait’s urban growth is primarily driven by factors other than population density. The most significant drivers, after historical land use patterns (evidence likelihood), are proximity to road infrastructure, coastline, and commercial areas. This finding challenges the conventional assumption that population growth and residential property prices are the main drivers of urban expansion, highlighting the unique dynamics of Kuwait’s urban development. In the same context, it is worth noting that the importance of the proximity to roadways during the period 1985–2005 was exceeded, during the 2005–2022 period, by that to the coastal line, revealing the importance of the selection of urbanization nearby the littoral line. The identification of key drivers of urban growth offers valuable insights for urban planners and policymakers. The projected urban expansion, particularly in the North-Northwestern and South-Southwestern regions, signals a significant transformation of Kuwait’s landscape.

The anticipated increase in urban areas from 819 km² in 2022–1,893 km² by 2100 highlights the scale of this transformation and the urgent need for sustainable urban development strategies. This expansion pattern, following a quasi-linear trend of approximately 12 km² per year, provides a clear trajectory for long-term planning. Equally significant is the projected growth in agricultural lands, from 477 km² in 2022 to approximately 897 km² by 2100. This expansion, while potentially beneficial for food security, raises questions about water resource management in an already water-scarce region. The study thus highlights the need for integrated land and water management strategies that can balance urban growth, agricultural expansion, and environmental sustainability.

The climate vulnerability analysis, based on the RCP 8.5 scenario, projects temperature increases of up to 17°C in urban and coastal regions by 2100. These changes pose potential risks to urban areas, including increased heat stress, challenges in water resource management, and potential infrastructure stress. Generally, the findings have significant implications for Kuwait’s urban planning policies and practices. They suggest a need for (1) Climate-resilient urban design, particularly in areas projected for high growth and high climate vulnerability (such as furnishing these areas with artificial and/or green drainage infrastructure in addition to enacting thermal insulation regulations), (2) Enhanced water management systems to address potential water scarcity issues, (3) Strategies to mitigate urban heat island effects, such as increasing urban green spaces with drought-resistant vegetation, (4) Investment in renewable energy sources to meet increased cooling demands sustainably, (5) Improved public health systems to deal with potential heat-related health issues, (6) Biodiversity conservation as a strategy to preserve and enhance biodiversity, particularly in vulnerable areas identified in the study. Furthermore, the research highlights the importance of balancing economic development with environmental sustainability. The projected growth in agricultural lands, alongside urban expansion, necessitates careful management to ensure food security without compromising environmental integrity. This study contributes significantly to the understanding of urban growth dynamics in rapidly developing, oil-rich nations with arid climates. It bridges the gap between urban growth modeling and climate change vulnerability assessment, providing a comprehensive framework for sustainable urban development in the face of global environmental change. However, the study acknowledges several limitations. Data constraints led to the exclusion of certain socio-economic variables, such as population growth trends, and political factors which may significantly influence urban development patterns. The satellite imagery used in the analysis may be subject to biases due to cloud cover and resolution constraints. Additionally, the model assumes a degree of stationarity in LULC drivers over time, which may not fully capture the dynamic nature of land use change. Furthermore, there are inherent uncertainties in long-term climate projections that could affect the accuracy of future LULC predictions. These limitations highlight the need for improved data integration and more sophisticated modeling approaches in future research. Future research could benefit from incorporating these additional variables and exploring alternative climate scenarios to provide a more nuanced understanding of potential future trajectories. More, The reliance on this single model for climate projections reflects a consistent framework aligning with FAO and RICCAR standards. Although using an ensemble of models can increase variability assessment, the EC-EARTH model was selected for its detailed projections in arid regions like Kuwait. Future work may explore multiple models to enhance scenario variability and refine predictions further. For future research, it is also proposed that advanced machine learning models, such as Convolutional Neural Networks, be updated to enhance spatial pattern recognition in LULC changes.

Additionally, it is suggested that real-time sensor data be integrated to improve prediction accuracy. More, broader range of climate scenarios must also be considered and applied to diverse regions to assess their generalizability and effectiveness. The findings and methodologies presented have broader applicability to other rapidly urbanizing regions, particularly in arid and semi-arid environments facing similar challenges. As Kuwait and similar nations continue to develop, the insights from this study can inform policies and practices that promote sustainable, resilient, and livable urban environments in the face of ongoing global environmental change.

## Supporting information

S1 Table Appendix 1. The attributes of the transition model (1985–2005). (DOCX)

S2 Table Appendix 1. The effect sizes of attributes in the transition model (1985–2005). (DOCX)

S3 Table Appendix 1. The attributes of the transition model (2005–2022). (DOCX)

S4 Table Appendix 1. The effect sizes of attributes in the transition model (2005–2022). (DOCX)

S1 Fig Appendix 2. (a) Elevation [78], (b) Slope, (c) Distance to industrial areas and quarries [79], (d) Distance to Parkings and Fuel Stations [79], (e) Distance to residential areas [79], (f) Distance to roadways [79]. (DOCX)

S2 Fig Appendix 2. (a) Distance to coastline, (b) Distance to waterways [79], (c) Distance to commercial areas [79], (d) Road line density, (e) Commercial property prices [73], (f) Residential property prices [73]. (DOCX)

S3 Fig Appendix 2. (a) Population Density for the year 1985 [77], (b) Population Density for the year 2005 [77], (c) Population Density for the year 2022 [77], (d) Point density of Parkings and Fuel Stations [78], (e) Evidence likelihood 1985–2005, (f) Evidence likelihood 2005–2022. (DOCX)

S4 Fig Appendix 2. Actual LULC maps for the years [75,76] (a) 1985 (b) 2005 (c) 2022 and (d) the predicted LULC map for the year 2022. (DOCX)
