# Supplementary material for: Urban morphology and climate vulnerability assessment in Kuwait: A spatio-temporal predictive analysis utilizing deep neural network-enhanced markov chain models for 2050 and 2100
Source: PLoS One. 2025 Aug 18;20(8):e0318604. doi: 10.1371/journal.pone.0318604 (PMC12360559; doi:10.1371/journal.pone.0318604)
Supplement: S1 Table — (DOCX) [file pone.0318604.s001.docx]

**Table S1** The attributes of the transition model (1985-2005)

| Model | Variables included | Accuracy (%) | Skill measure |
| --- | --- | --- | --- |
| With all variables | All variables | 78.64 | 0.7597 |
| Step 1 | All variables except Residential property prices | 78.66 | 0.7599 |
| Step 2 | All variables except:   - Residential property prices - Point density of Parkings and Fuel stations | 78.66 | 0.76 |
| Step 3 | All variables except:   - Residential property prices - Point density of Parkings and Fuel stations - Topographic slope | 78.66 | 0.76 |
| Step 4 | All variables except:   - Residential property prices - Point density of Parkings and Fuel stations - Topographic slope - Line density of roadways | 78.66 | 0.76 |
| Step 5 | - Population Density 1985 - Population Density 2005 - Distance to residential areas - Distance to Waterways - Elevation - Distance to Coastline - Distance to Commercial areas - Distance to Industrial areas - Distance to Parkings and Fuel stations - Distance to Roadways - Population Density 2022 - Evidence Likelihood 1985-2005 | 78.65 | 0.7598 |
| Step 6 | - Population Density 2005 - Distance to residential areas - Distance to Waterways - Elevation - Distance to Coastline - Distance to Commercial areas - Distance to Industrial areas - Distance to Parkings and Fuel stations - Distance to Roadways - Population Density 2022 - Evidence Likelihood 1985-2005 | 78.65 | 0.7598 |
| Step 7 | - Distance to residential areas - Distance to Waterways - Elevation - Distance to Coastline - Distance to Commercial areas - Distance to Industrial areas - Distance to Parkings and Fuel stations - Distance to Roadways - Population Density 2022 - Evidence Likelihood 1985-2005 | 78.43 | 0.7573 |

**Table S1 (Continued)** The attributes of the transition model (1985-2005)

| Model | Variables included | Accuracy (%) | Skill measure |
| --- | --- | --- | --- |
| Step 8 | - Elevation - Distance to Waterways - Distance to Coastline - Distance to Commercial areas - Distance to Industrial areas - Distance to Parkings and Fuel stations - Distance to Roadways - Population Density 2022 - Evidence Likelihood 1985-2005 | 77.11 | 0.7425 |
| Step 9 | - Elevation - Distance to Coastline - Distance to Commercial areas - Distance to Industrial areas - Distance to Parkings and Fuel stations - Distance to Roadways - Population Density 2022 - Evidence Likelihood 1985-2005 | 75.82 | 0.7279 |
| Step 10 | - Distance to Coastline - Distance to Commercial areas - Distance to Industrial areas - Distance to Parkings and Fuel stations - Distance to Roadways - Population Density 2022 - Evidence Likelihood 1985-2005 | 74.46 | 0.7127 |
| Step 11 | - Distance to Coastline - Distance to Commercial areas - Distance to Industrial areas - Distance to Roadways - Population Density 2022 - Evidence Likelihood 1985-2005 | 73.52 | 0.7021 |
| Step 12 | - Distance to Coastline - Distance to Commercial areas - Distance to Roadways - Population Density 2022 - Evidence Likelihood 1985-2005 | 71.26 | 0.6766 |
| Step 13 | - Distance to Coastline - Distance to Commercial areas - Distance to Roadways - Evidence Likelihood 1985-2005 | 68.99 | 0.6511 |
| Step 14 | - Distance to Coastline - Distance to Roadways - Evidence Likelihood 1985-2005 | 58.7 | 0.5353 |
| Step 15 | - Distance to Coastline - Evidence Likelihood 1985-2005 | 45.78 | 0.3901 |
| Step 16 | Evidence Likelihood 1985-2005 | 33.35 | 0.2502 |
