# Supplementary material for: Urban morphology and climate vulnerability assessment in Kuwait: A spatio-temporal predictive analysis utilizing deep neural network-enhanced markov chain models for 2050 and 2100
Source: PLoS One. 2025 Aug 18;20(8):e0318604. doi: 10.1371/journal.pone.0318604 (PMC12360559; doi:10.1371/journal.pone.0318604)
Supplement: S2 Table — (DOCX) [file pone.0318604.s002.docx]

**Table S2** The effect sizes of attributes in the transition model (1985-2005)

| Rank | Variable | Accuracy (%) | Skill measure |
| --- | --- | --- | --- |
| 1 | Evidence Likelihood 1985-2005 | 33.35 | 0.2502 |
| 2 | Distance to Roadways | 12.92 | 0.1452 |
| 3 | Distance to Coastline | 12.43 | 0.1399 |
| 4 | Distance to Commercial areas | 10.29 | 0.1158 |
| 5 | Population Density 2022 | 2.27 | 0.0255 |
| 6 | Distance to Industrial areas | 2.26 | 0.0255 |
| 7 | Elevation | 1.36 | 0.0152 |
| 8 | Distance to residential areas | 1.32 | 0.0148 |
| 9 | Distance to Waterways | 1.29 | 0.0146 |
| 10 | Distance to Parkings and Fuel stations | 0.94 | 0.0106 |
| 11 | Population Density 2005 | 0.22 | 0.0025 |
| 12 | Commercial property prices | 0.01 | 0.0002 |
| 13 | Point density of Parkings and Fuel stations | 0 | -0.0001 |
| 14 | Slope | 0 | 0 |
| 15 | Line density of roadways | 0 | 0 |
| 16 | Population Density 1985 | 0 | 0 |
| 17 | Residential property prices | -0.02 | -0.0002 |
