# Supplementary material for: Urban morphology and climate vulnerability assessment in Kuwait: A spatio-temporal predictive analysis utilizing deep neural network-enhanced markov chain models for 2050 and 2100
Source: PLoS One. 2025 Aug 18;20(8):e0318604. doi: 10.1371/journal.pone.0318604 (PMC12360559; doi:10.1371/journal.pone.0318604)
Supplement: S3 Table — (DOCX) [file pone.0318604.s003.docx]

**Table S3** The attributes of the transition model (2005-2022)

| Model | Variables included | Accuracy (%) | Skill measure |
| --- | --- | --- | --- |
| With all variables | All variables | 76.61 | 0.7369 |
| Step 1 | All variables except Residential property prices | 76.64 | 0.7372 |
| Step 2 | All variables except:   - Residential property prices - Point density of Parkings and Fuel stations | 76.64 | 0.7373 |
| Step 3 | All variables except:   - Residential property prices - Point density of Parkings and Fuel stations - Topographic slope | 76.65 | 0.7373 |
| Step 4 | All variables except:   - Residential property prices - Point density of Parkings and Fuel stations - Topographic slope - Line density of roadways | 76.64 | 0.7373 |
| Step 5 | - Elevation - Distance to Waterways - Distance to Coastline - Distance to Commercial areas - Distance to residential areas - Distance to Parkings and Fuel stations - Distance to Roadways - Distance to Industrial areas - Population Density 1985 - Population Density 2005 - Population Density 2022 - Evidence Likelihood 1985-2005 | 76.64 | 0.7372 |
| Step 6 | - Elevation - Distance to Waterways - Distance to Coastline - Distance to Commercial areas - Distance to residential areas - Distance to Parkings and Fuel stations - Distance to Roadways - Distance to Industrial areas - Population Density 2005 - Population Density 2022 - Evidence Likelihood 1985-2005 | 76.59 | 0.7366 |
| Step 7 | - Elevation - Distance to Waterways - Distance to Coastline - Distance to Commercial areas - Distance to residential areas - Distance to Parkings and Fuel stations - Distance to Roadways - Distance to Industrial areas - Population Density 2022 - Evidence Likelihood 1985-2005 | 76.49 | 0.7355 |

**Table S3 (continued)** The attributes of the transition model (2005-2022)

| Model | Variables included | Accuracy (%) | Skill measure |
| --- | --- | --- | --- |
| Step 8 | - Elevation - Distance to Waterways - Distance to Coastline - Distance to Commercial areas - Distance to residential areas - Distance to Parkings and Fuel stations - Distance to Roadways - Distance to Industrial areas - Evidence Likelihood 1985-2005 | 75.97 | 0.7297 |
| Step 9 | - Elevation - Distance to Coastline - Distance to Commercial areas - Distance to residential areas - Distance to Parkings and Fuel stations - Distance to Roadways - Distance to Waterways - Evidence Likelihood 1985-2005 | 75.28 | 0.7219 |
| Step 10 | - Elevation - Distance to Coastline - Distance to Commercial areas - Distance to Parkings and Fuel stations - Distance to Roadways - Distance to Waterways - Evidence Likelihood 1985-2005 | 74.19 | 0.7096 |
| Step 11 | - Distance to Coastline - Distance to Commercial areas - Distance to Waterways - Distance to Roadways - Distance to Parkings and Fuel stations - Evidence Likelihood 1985-2005 | 72.65 | 0.6923 |
| Step 12 | - Distance to Coastline - Distance to Commercial areas - Distance to Roadways - Distance to Parkings and Fuel stations - Evidence Likelihood 1985-2005 | 71.68 | 0.6814 |
| Step 13 | - Distance to Coastline - Distance to Commercial areas - Distance to Roadways - Evidence Likelihood 1985-2005 | 67.51 | 0.6345 |
| Step 14 | - Distance to Roadways - Distance to Coastline - Evidence Likelihood 1985-2005 | 59.03 | 0.5391 |
| Step 15 | - Distance to Coastline - Evidence Likelihood 1985-2005 | 47.06 | 0.4044 |
| Step 16 | Evidence Likelihood 1985-2005 | 33.25 | 0.2490 |
