# Supplementary material for: Urban morphology and climate vulnerability assessment in Kuwait: A spatio-temporal predictive analysis utilizing deep neural network-enhanced markov chain models for 2050 and 2100
Source: PLoS One. 2025 Aug 18;20(8):e0318604. doi: 10.1371/journal.pone.0318604 (PMC12360559; doi:10.1371/journal.pone.0318604)
Supplement: S4 Table — (DOCX) [file pone.0318604.s004.docx]

**Table S4** The effect sizes of attributes in the transition model (2005-2022)

| Rank | Variable | Accuracy (%) | Skill measure |
| --- | --- | --- | --- |
| 1 | Evidence Likelihood 2005-2022 | 33.25 | 0.249 |
| 2 | Distance to Coastline | 13.81 | 0.1554 |
| 3 | Distance to Roadways | 11.97 | 0.1347 |
| 4 | Distance to Commercial areas | 8.48 | 0.0954 |
| 5 | Distance to Parkings and Fuel stations | 4.17 | 0.0469 |
| 6 | Elevation | 1.54 | 0.0173 |
| 7 | Distance to residential areas | 1.09 | 0.0123 |
| 8 | Distance to Waterways | 0.97 | 0.0109 |
| 9 | Distance to Industrial areas | 0.69 | 0.0078 |
| 10 | Population Density 2022 | 0.52 | 0.0058 |
| 11 | Population Density 2005 | 0.1 | 0.0011 |
| 12 | Population Density 1985 | 0.05 | 0.0006 |
| 13 | Line density of roadways | 0.01 | 0 |
| 14 | Point density of Parkings and Fuel stations | 0 | -0.0001 |
| 15 | Commercial property prices | 0 | 0.0001 |
| 16 | Slope | -0.01 | 0 |
| 17 | Residential property prices | -0.03 | -0.0003 |
