# Supplementary material for: Urban morphology and climate vulnerability assessment in Kuwait: A spatio-temporal predictive analysis utilizing deep neural network-enhanced markov chain models for 2050 and 2100
Source: PLoS One. 2025 Aug 18;20(8):e0318604. doi: 10.1371/journal.pone.0318604 (PMC12360559; doi:10.1371/journal.pone.0318604)
Supplement: S1 Fig — (DOCX) [file pone.0318604.s005.docx]

| 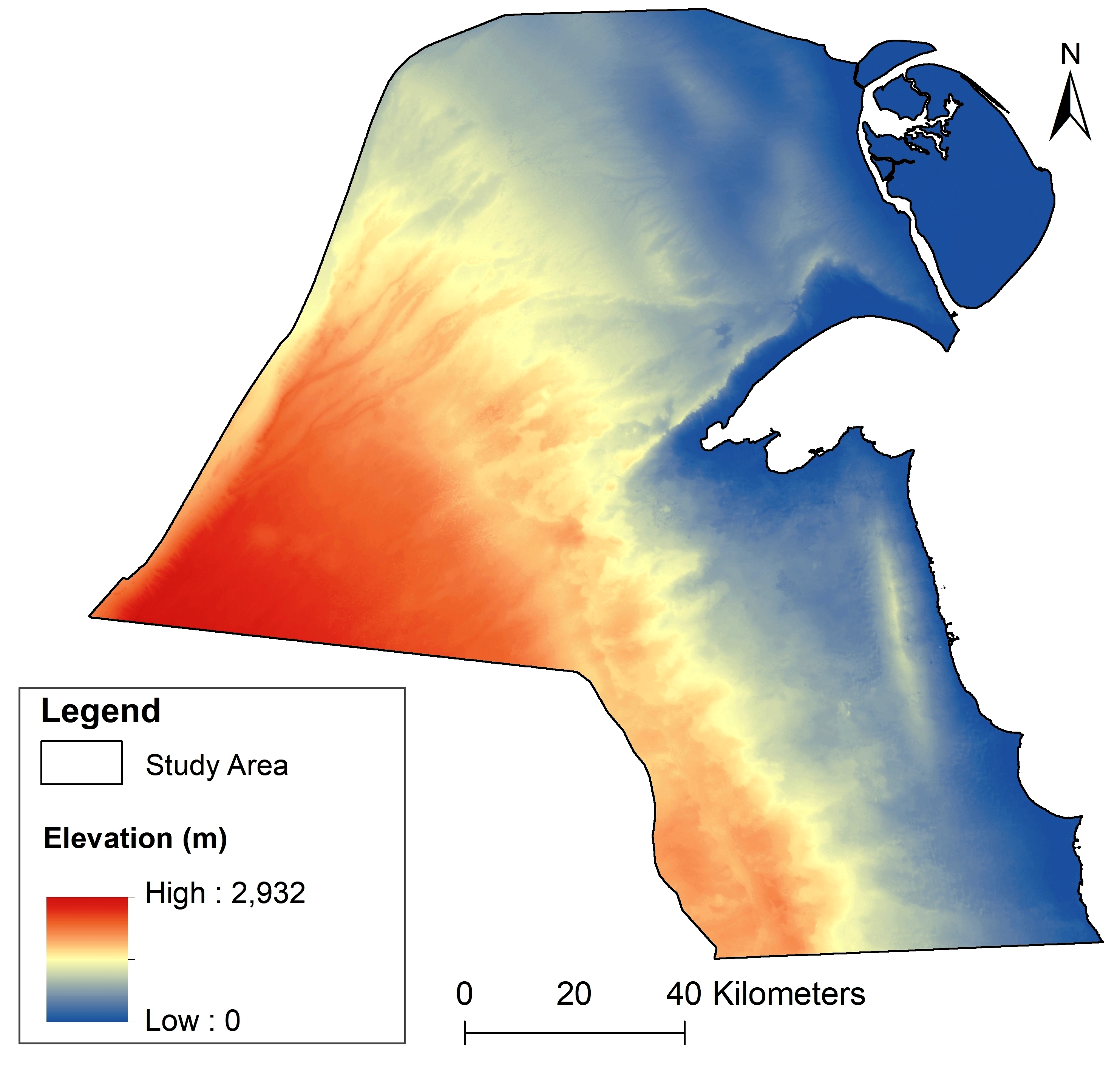 | 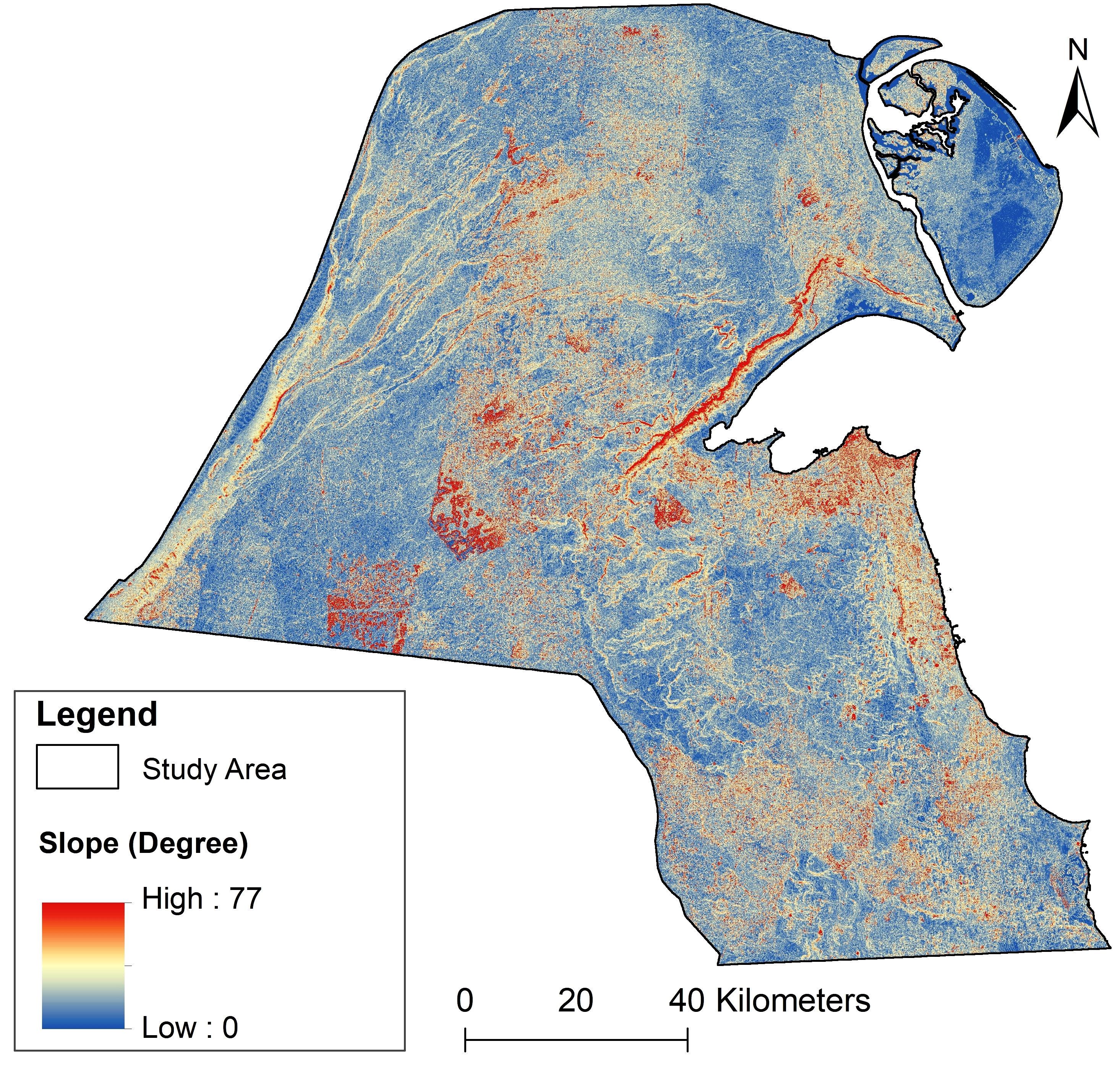 |
| --- | --- |
| **(a)** | **(b)** |
| 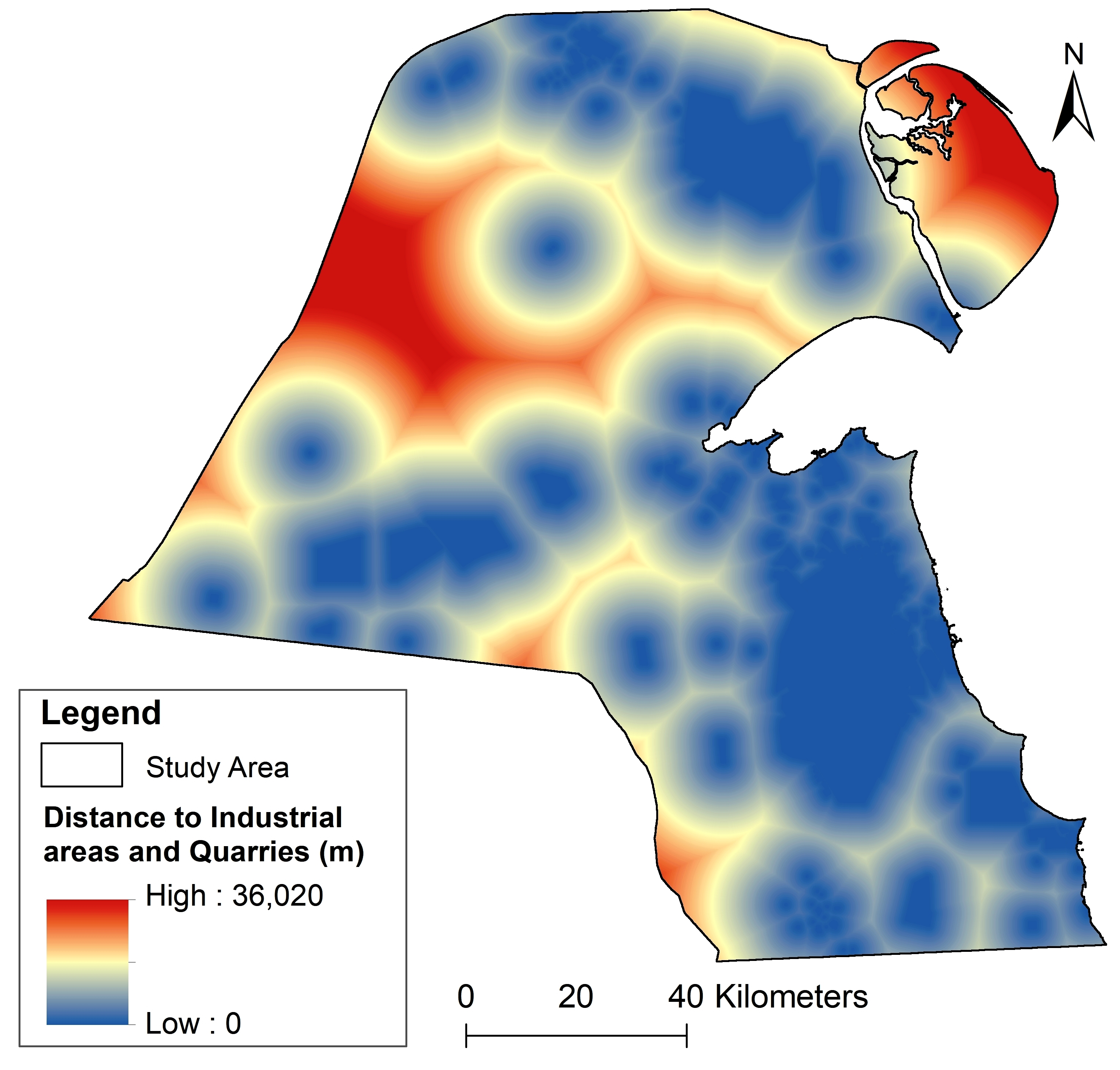 | 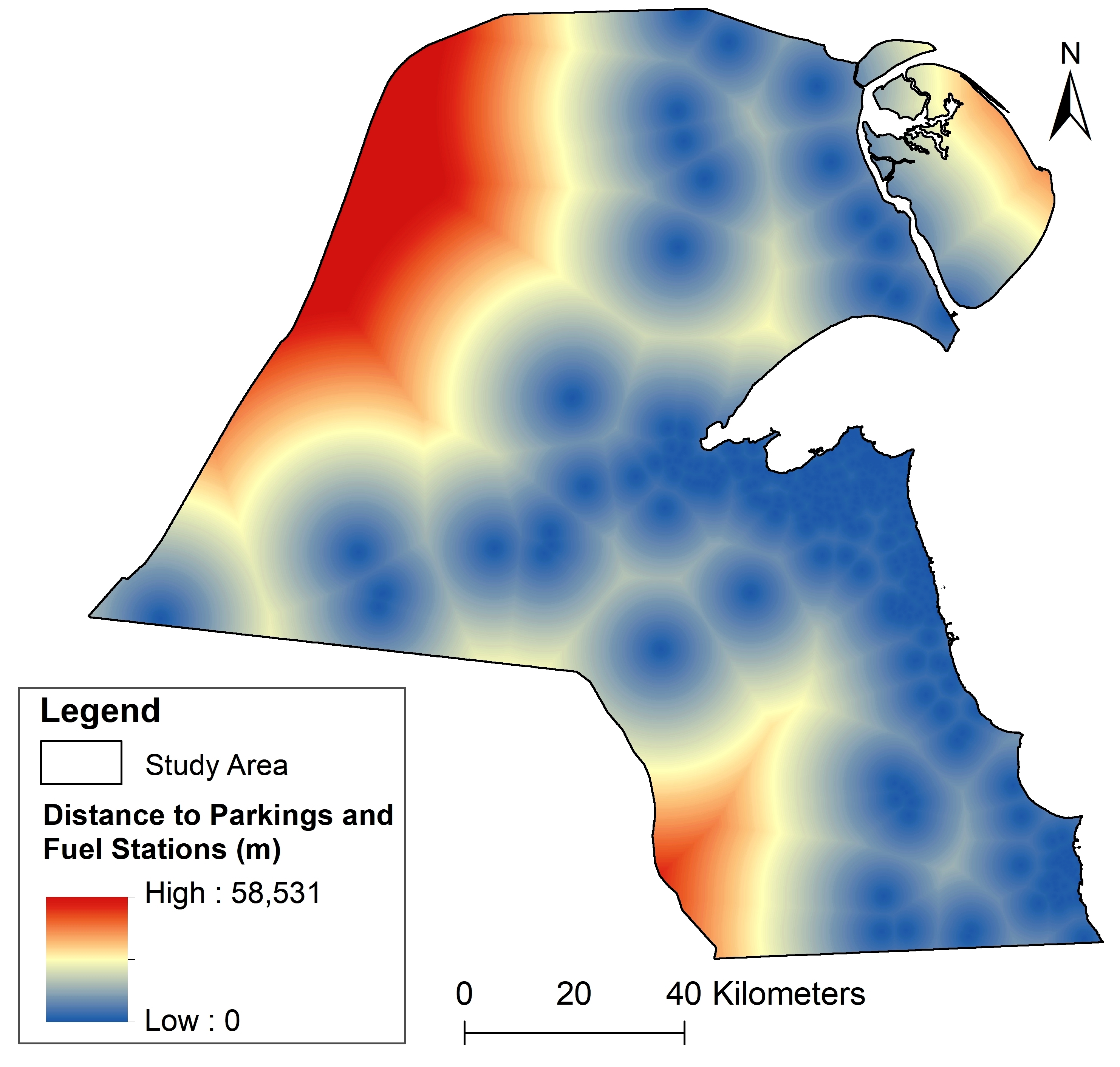 |
| **(c)** | **(d)** |
| 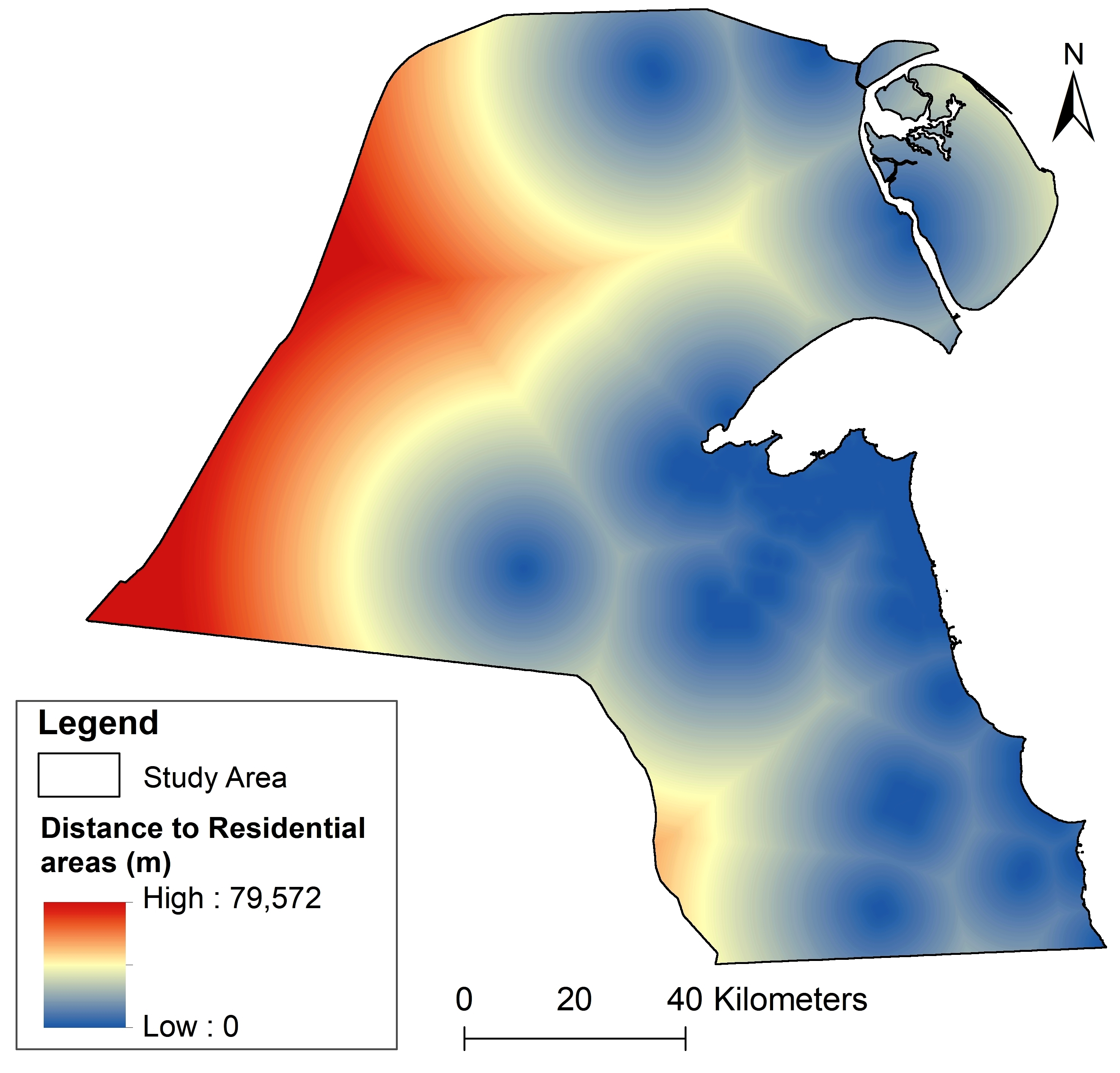 | 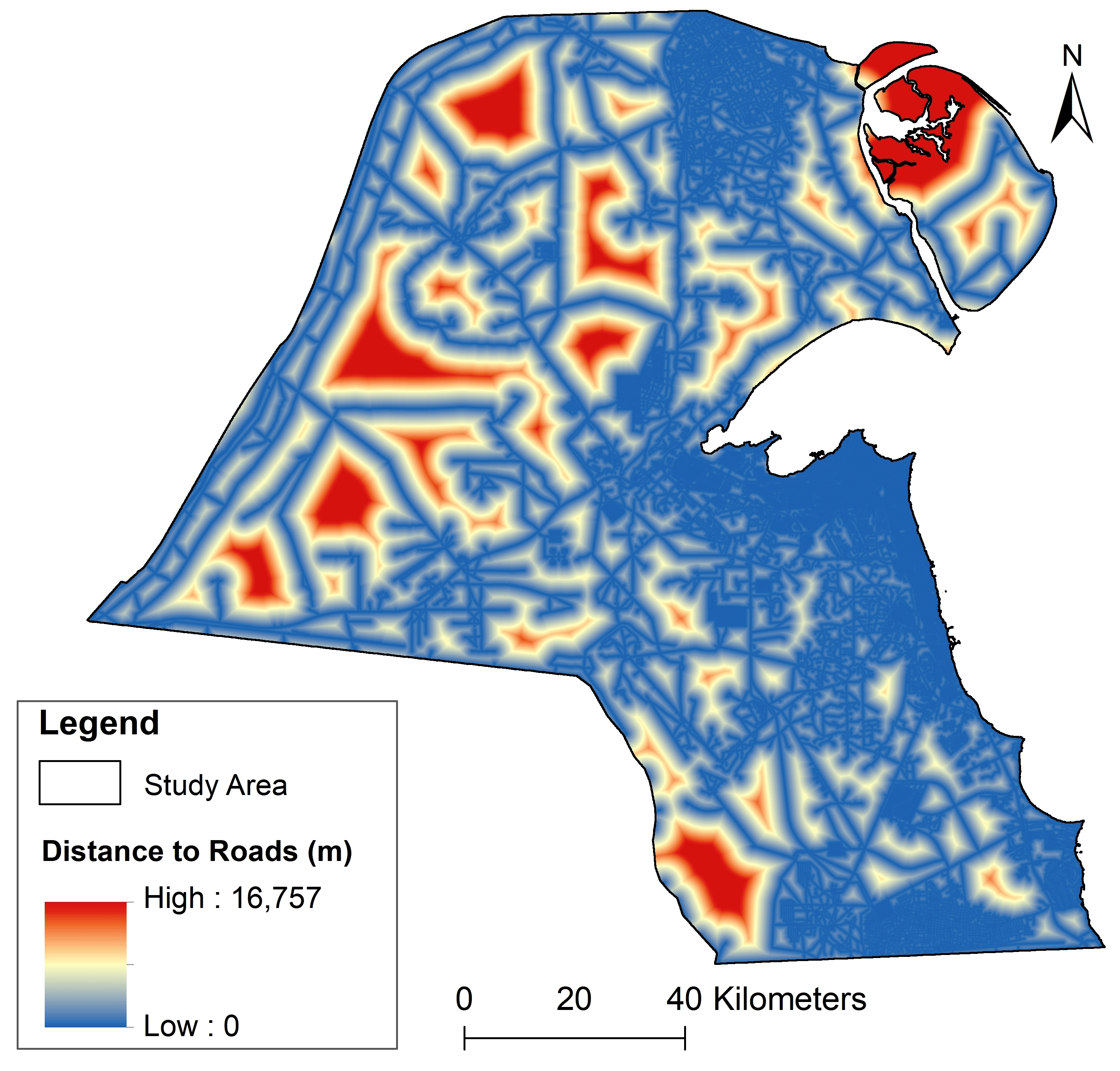 |
| **(e)** | **(f)** |

**Fig. S1** (a) Elevation [78], (b) Slope, (c) Distance to industrial areas and quarries [79], (d) Distance to Parkings and Fuel Stations [79], (e) Distance to residential areas [79], (f) Distance to roadways [79]
