# Supplementary material for: Urban morphology and climate vulnerability assessment in Kuwait: A spatio-temporal predictive analysis utilizing deep neural network-enhanced markov chain models for 2050 and 2100
Source: PLoS One. 2025 Aug 18;20(8):e0318604. doi: 10.1371/journal.pone.0318604 (PMC12360559; doi:10.1371/journal.pone.0318604)
Supplement: S2 Fig — (DOCX) [file pone.0318604.s006.docx]

| 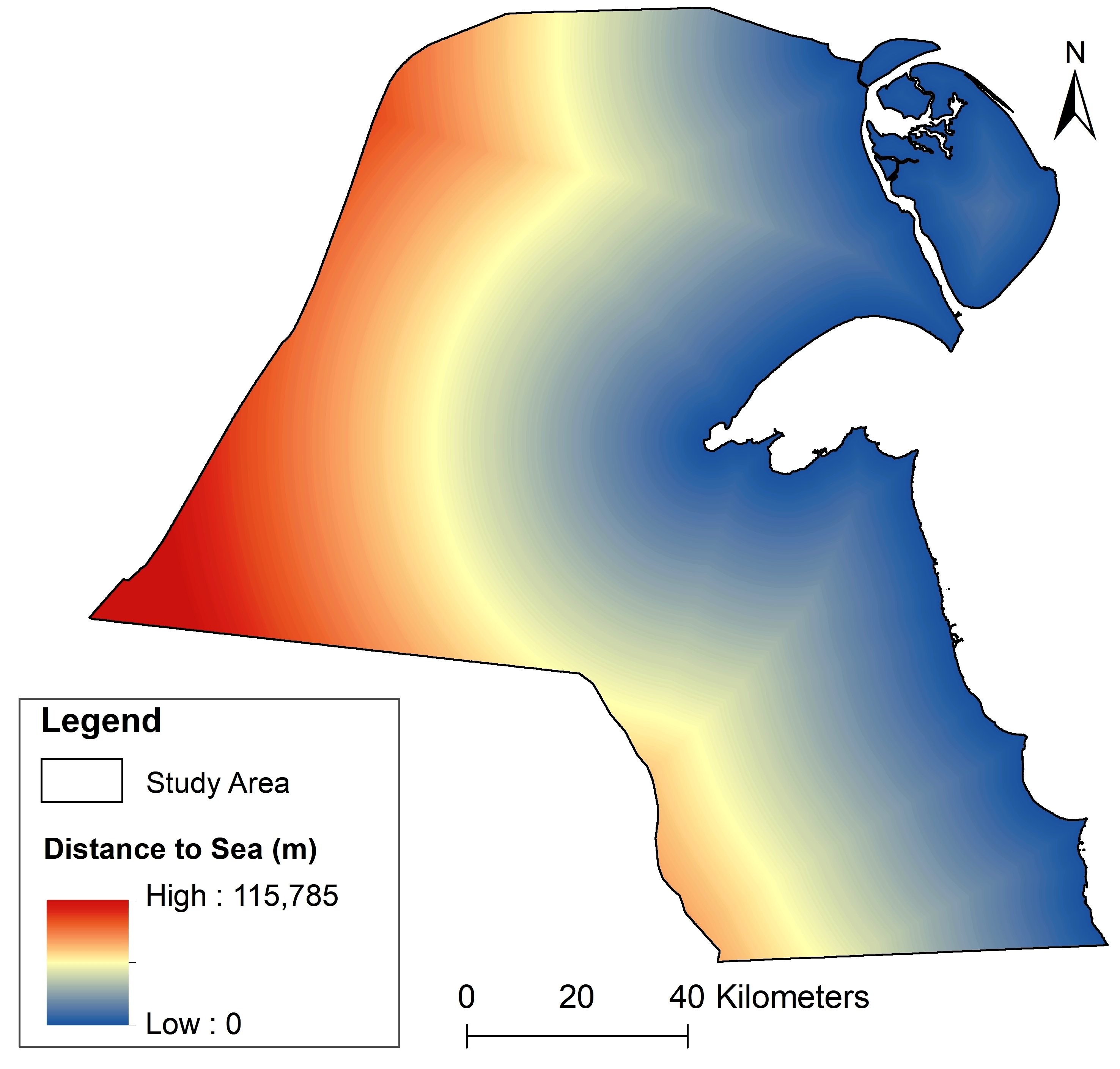 | 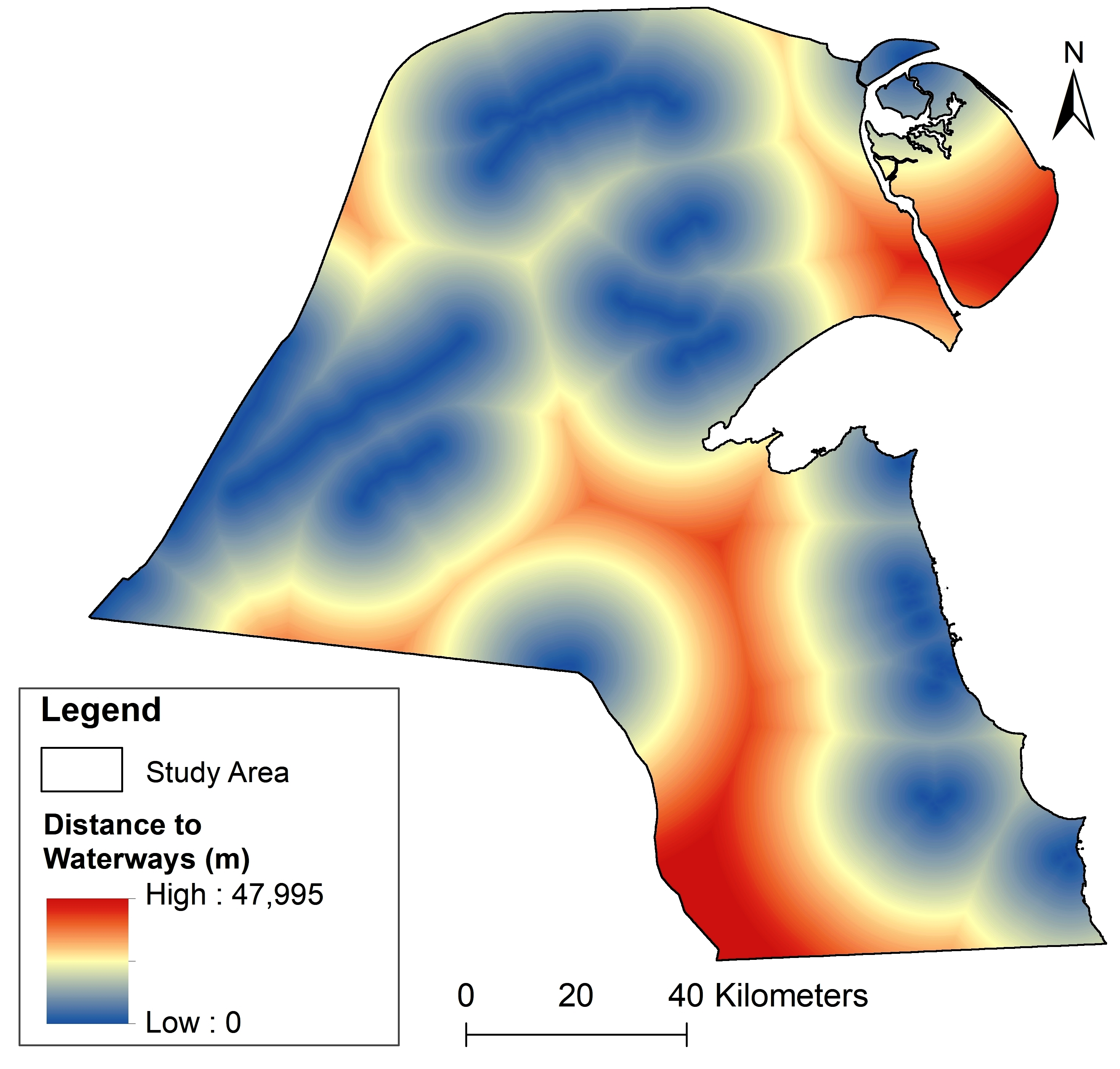 |
| --- | --- |
| **(a)** | **(b)** |
| 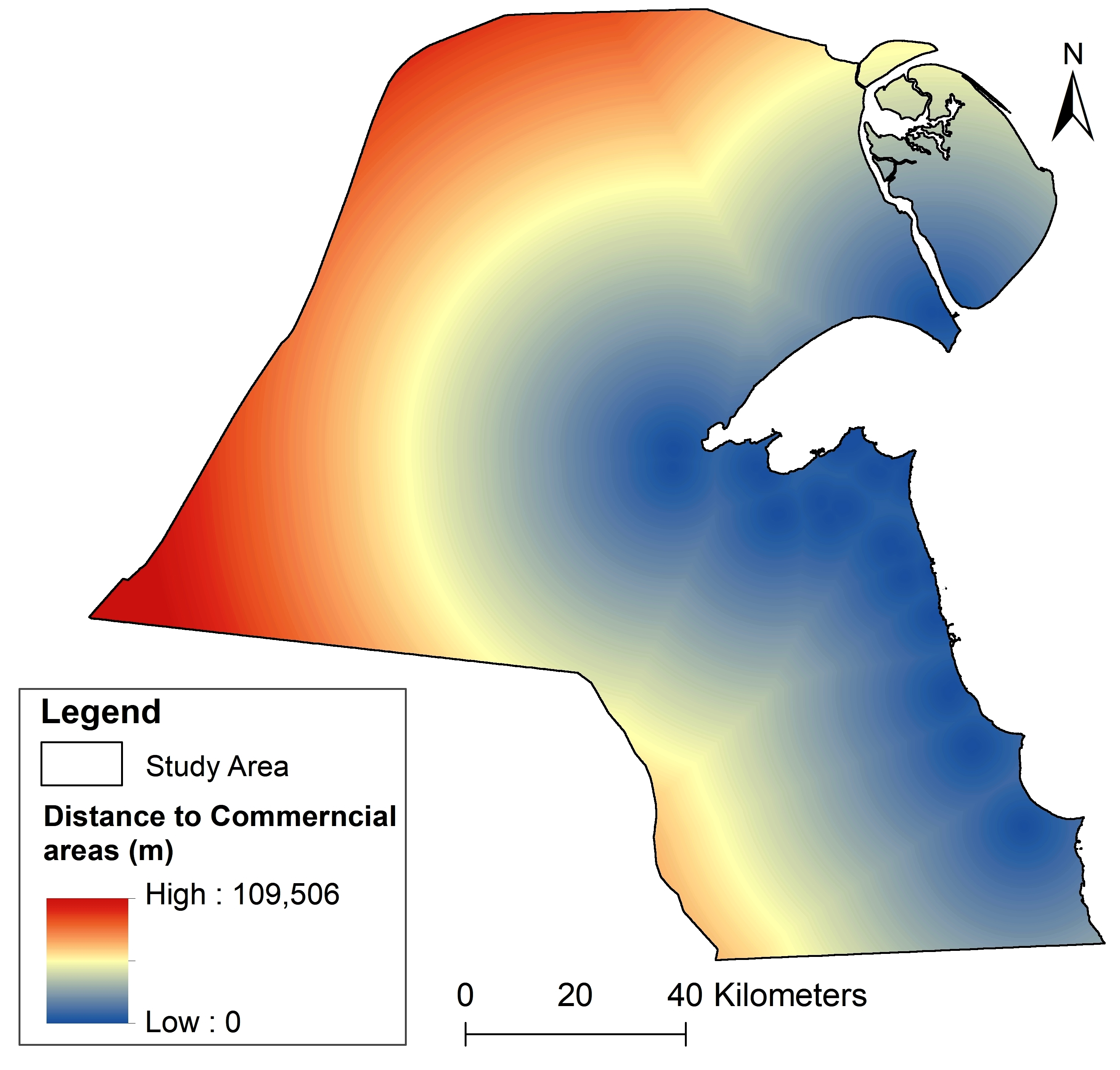 | 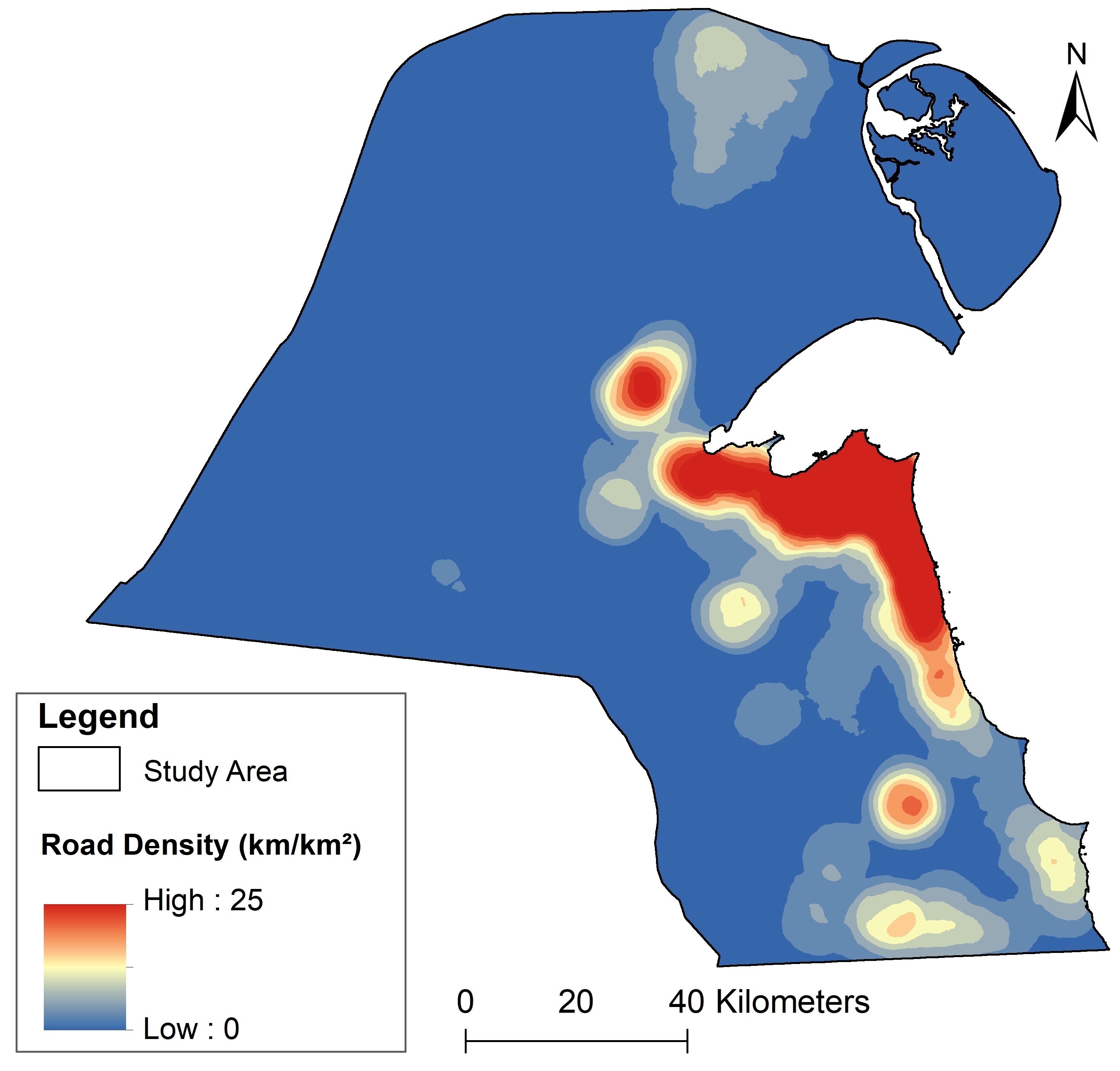 |
| **(c)** | **(d)** |
| 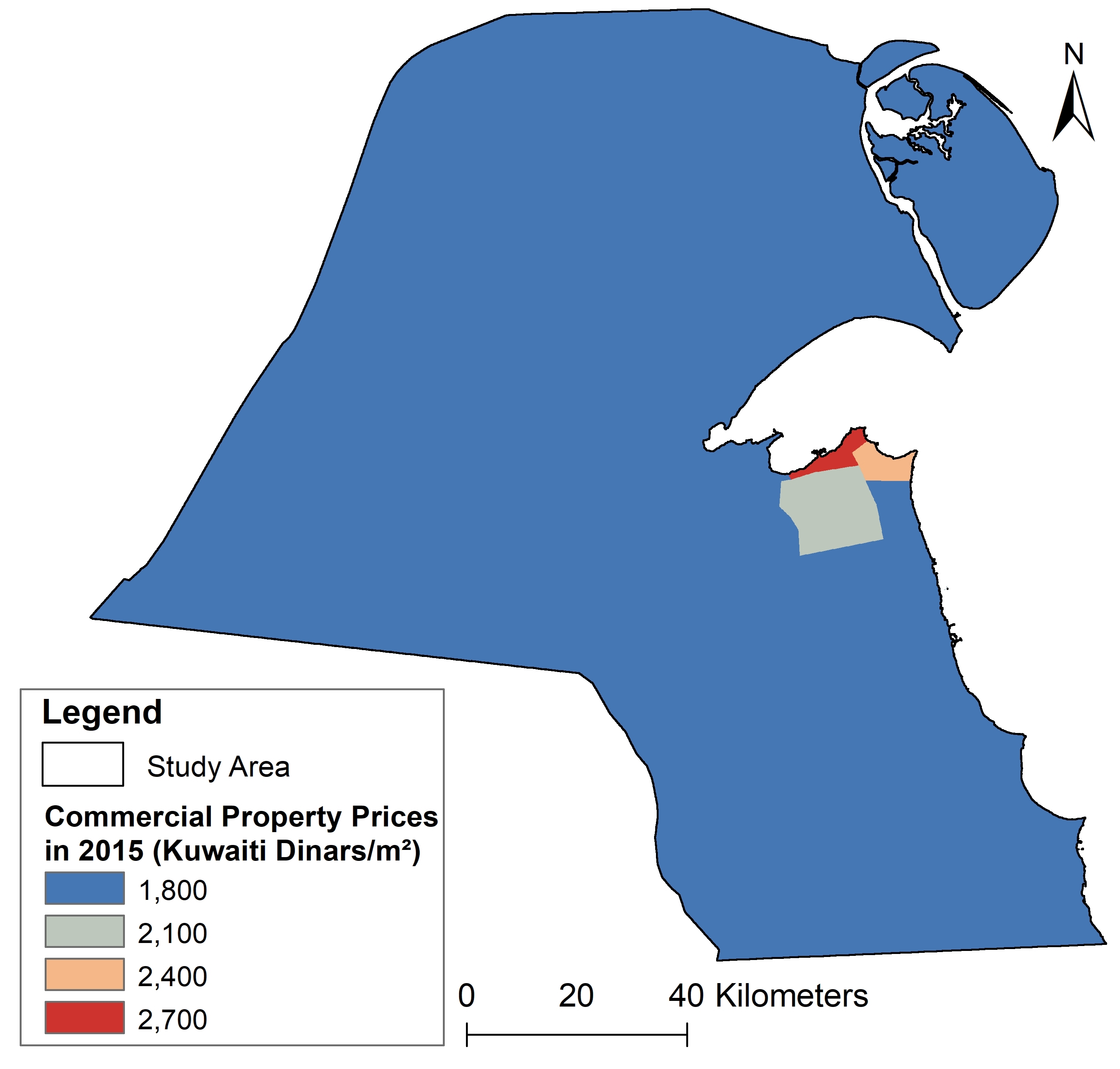 | 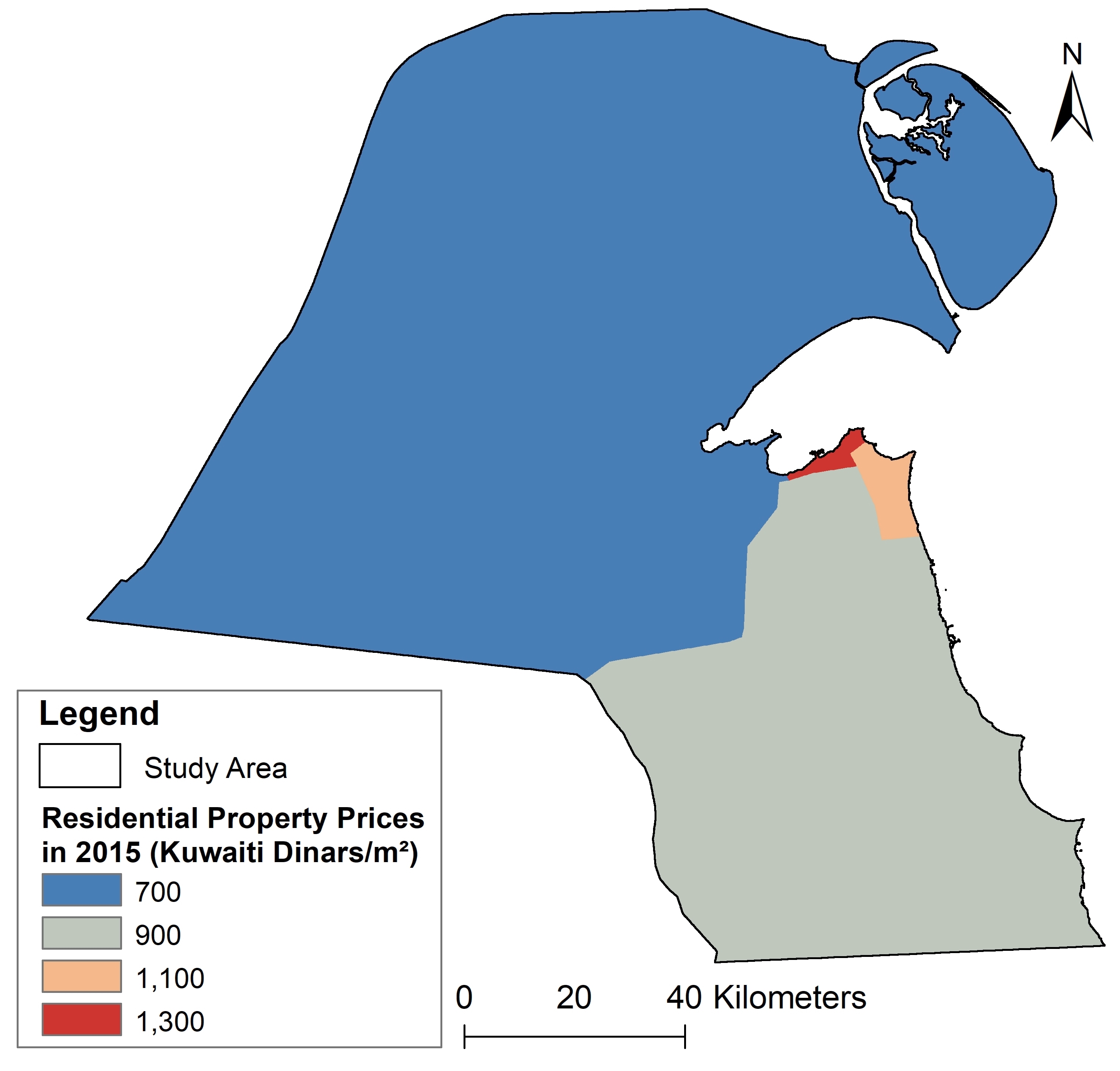 |
| **(e)** | **(f)** |

**Fig. S2** (a) Distance to coastline, (b) Distance to waterways [79], (c) Distance to commercial areas [79], (d) Road line density, (e) Commercial property prices [73], (f) Residential property prices [73]
