# Supplementary material for: Urban morphology and climate vulnerability assessment in Kuwait: A spatio-temporal predictive analysis utilizing deep neural network-enhanced markov chain models for 2050 and 2100
Source: PLoS One. 2025 Aug 18;20(8):e0318604. doi: 10.1371/journal.pone.0318604 (PMC12360559; doi:10.1371/journal.pone.0318604)
Supplement: S3 Fig — (DOCX) [file pone.0318604.s007.docx]

| 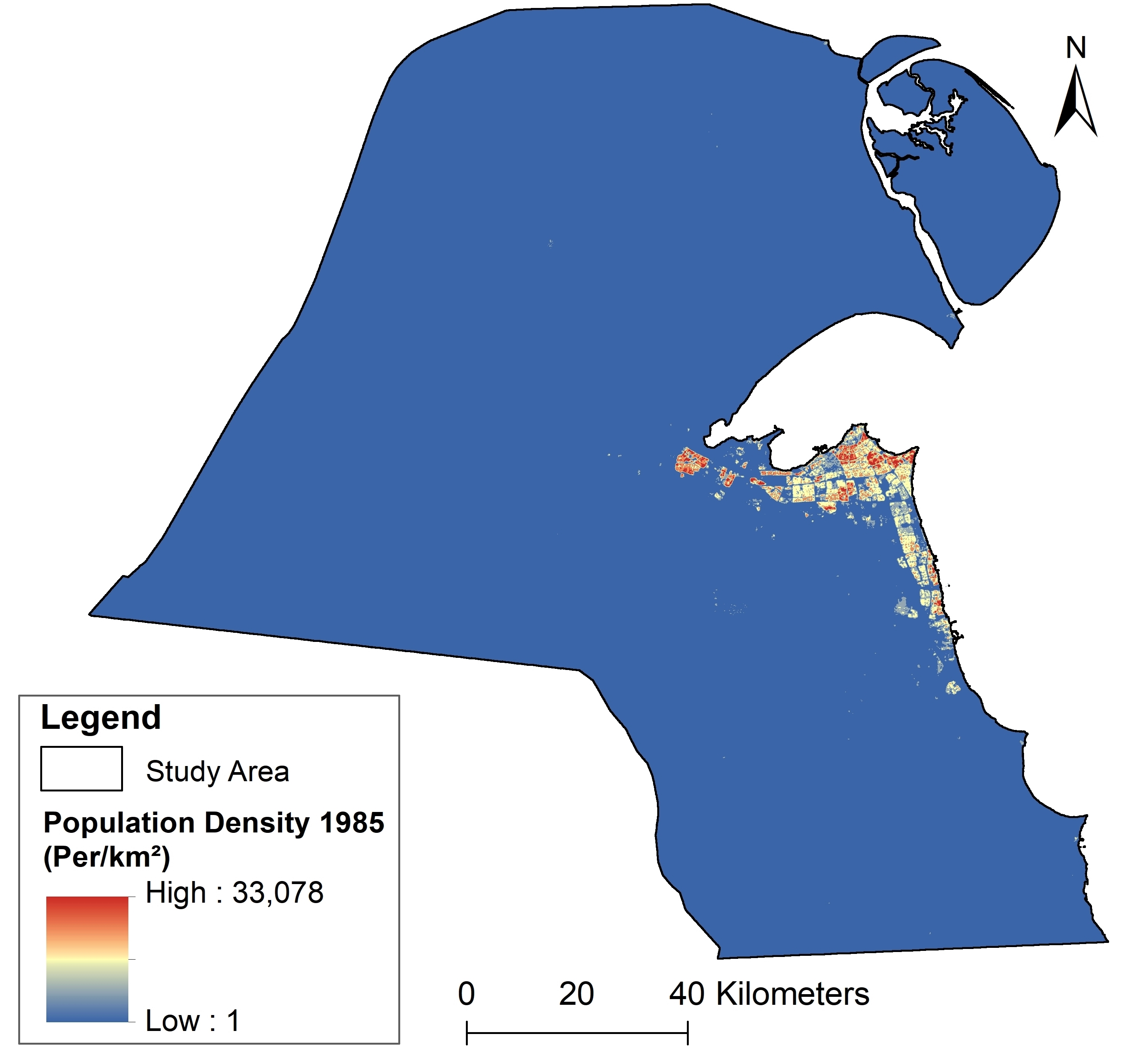 | 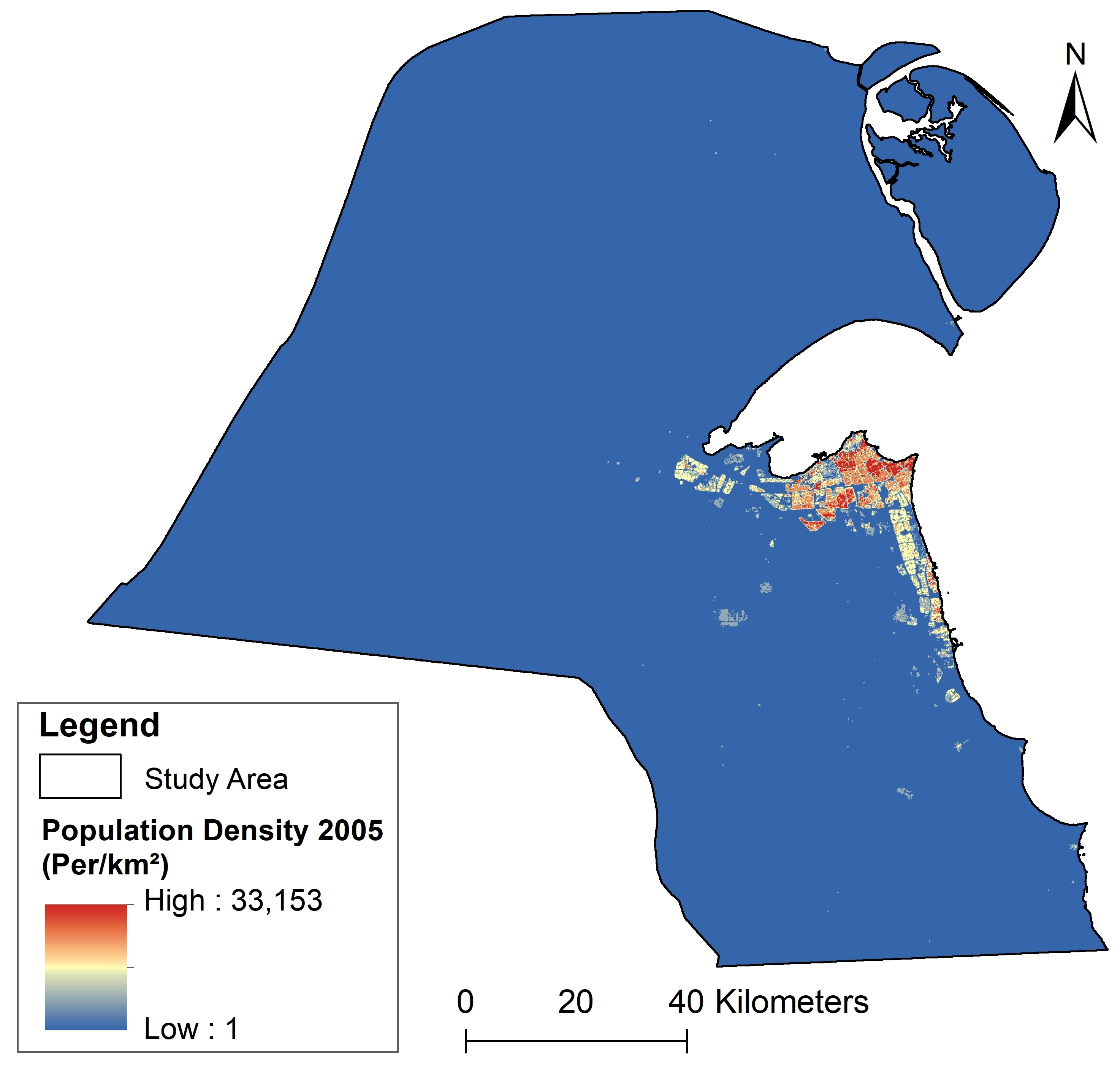 |
| --- | --- |
| **(a)** | **(b)** |
| 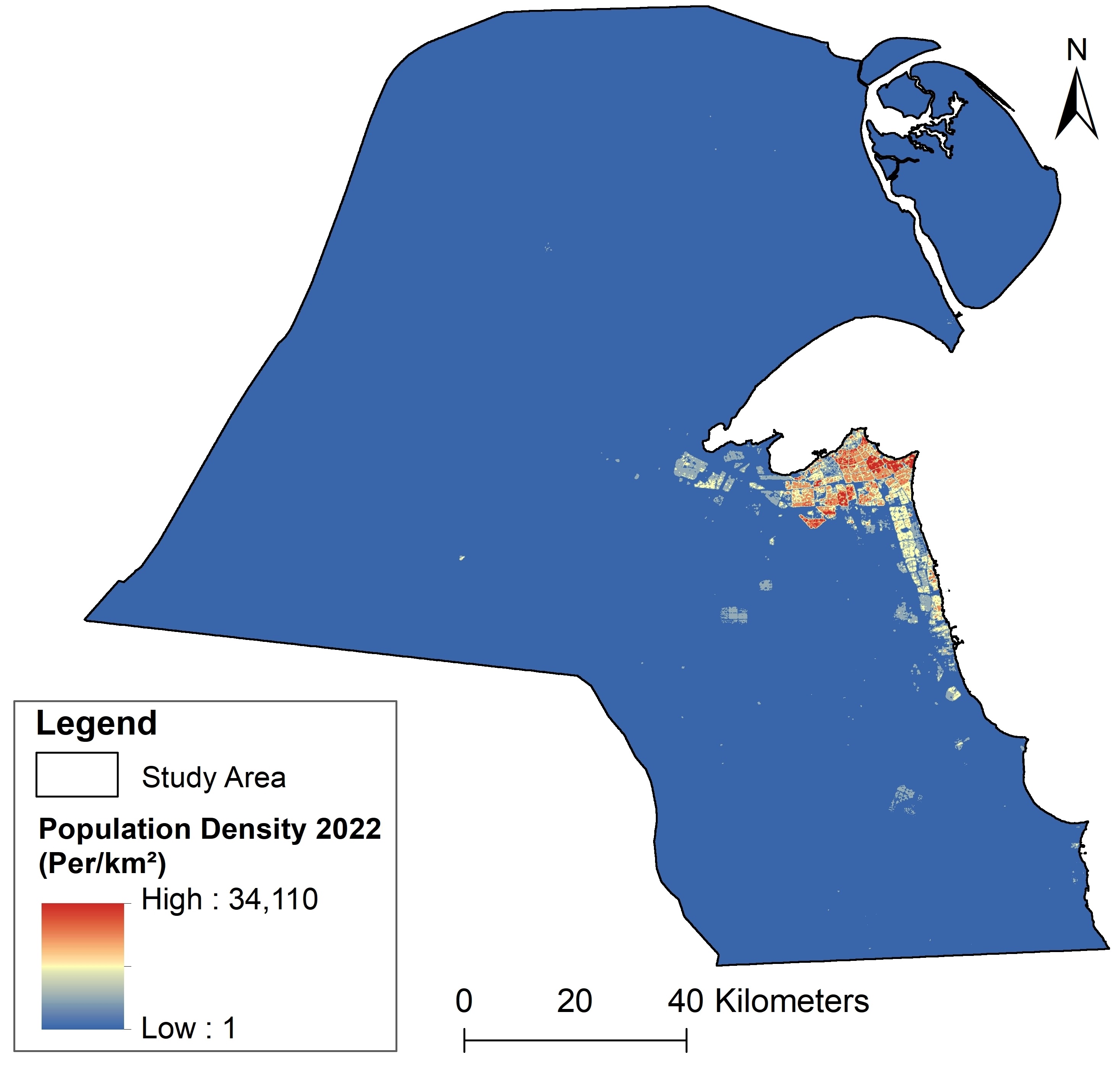 | 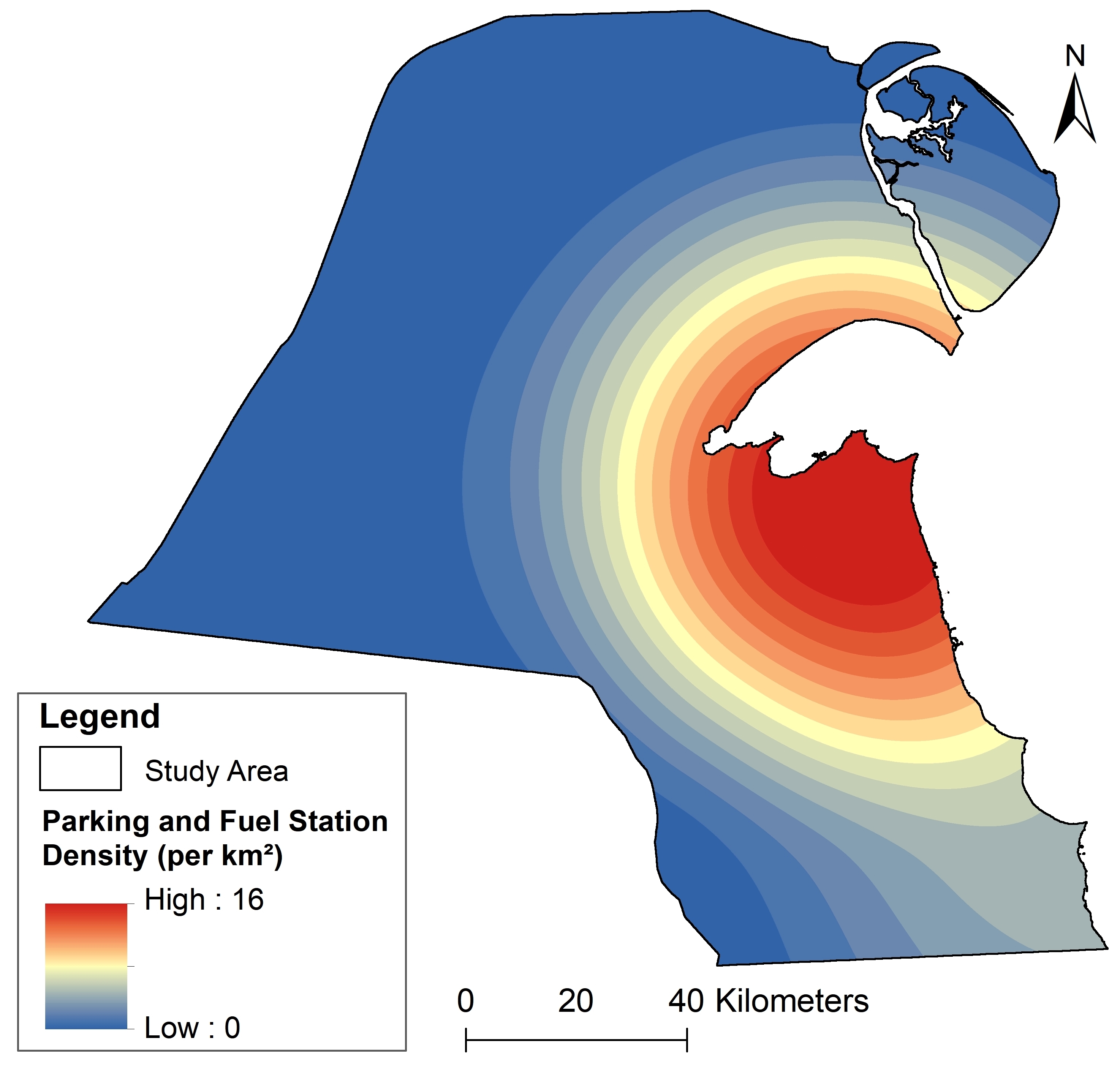 |
| **(c)** | **(d)** |
| 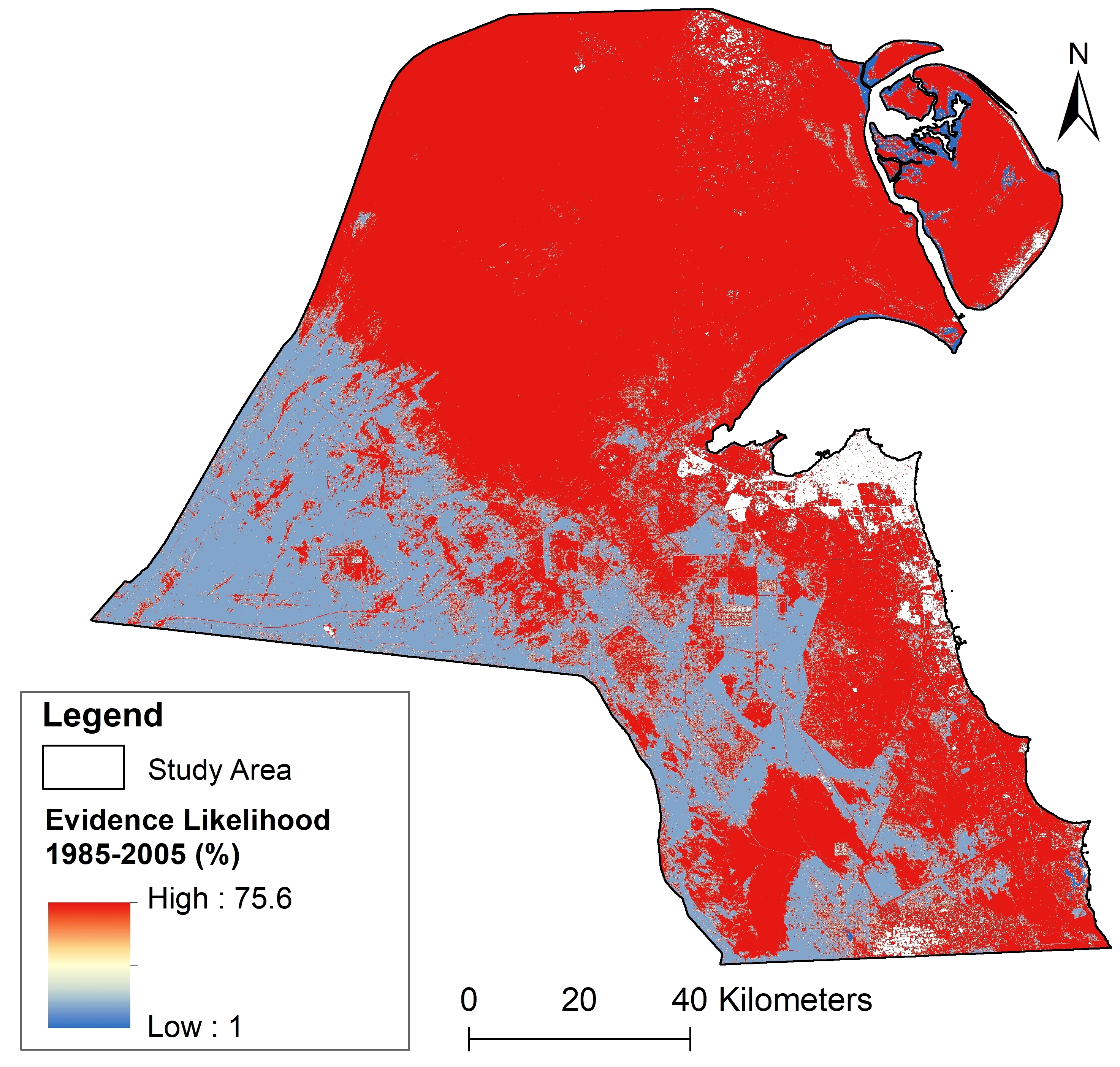 | 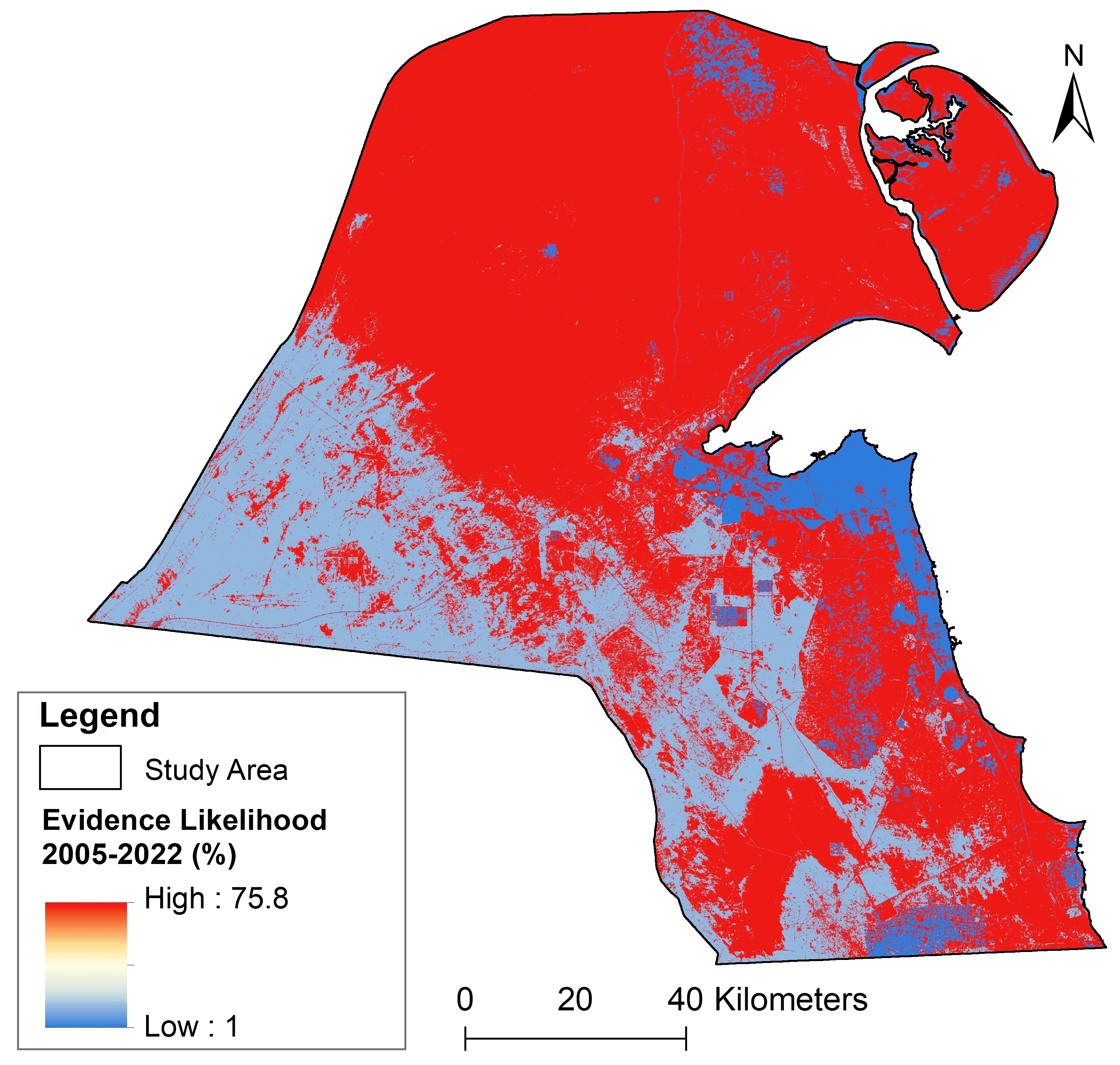 |
| **(e)** | **(f)** |

**Fig. S3** (a) Population Density for the year 1985 [77], (b) Population Density for the year 2005 [77], (c) Population Density for the year 2022 [77], (d) Point density of Parkings and Fuel Stations [78], (e) Evidence likelihood 1985-2005, (f) Evidence likelihood 2005-2022
