# Supplementary figures and images for: Urban morphology and climate vulnerability assessment in Kuwait: A spatio-temporal predictive analysis utilizing deep neural network-enhanced markov chain models for 2050 and 2100
Source: PLoS One. 2025 Aug 18;20(8):e0318604. doi: 10.1371/journal.pone.0318604 (PMC12360559; doi:10.1371/journal.pone.0318604)

| 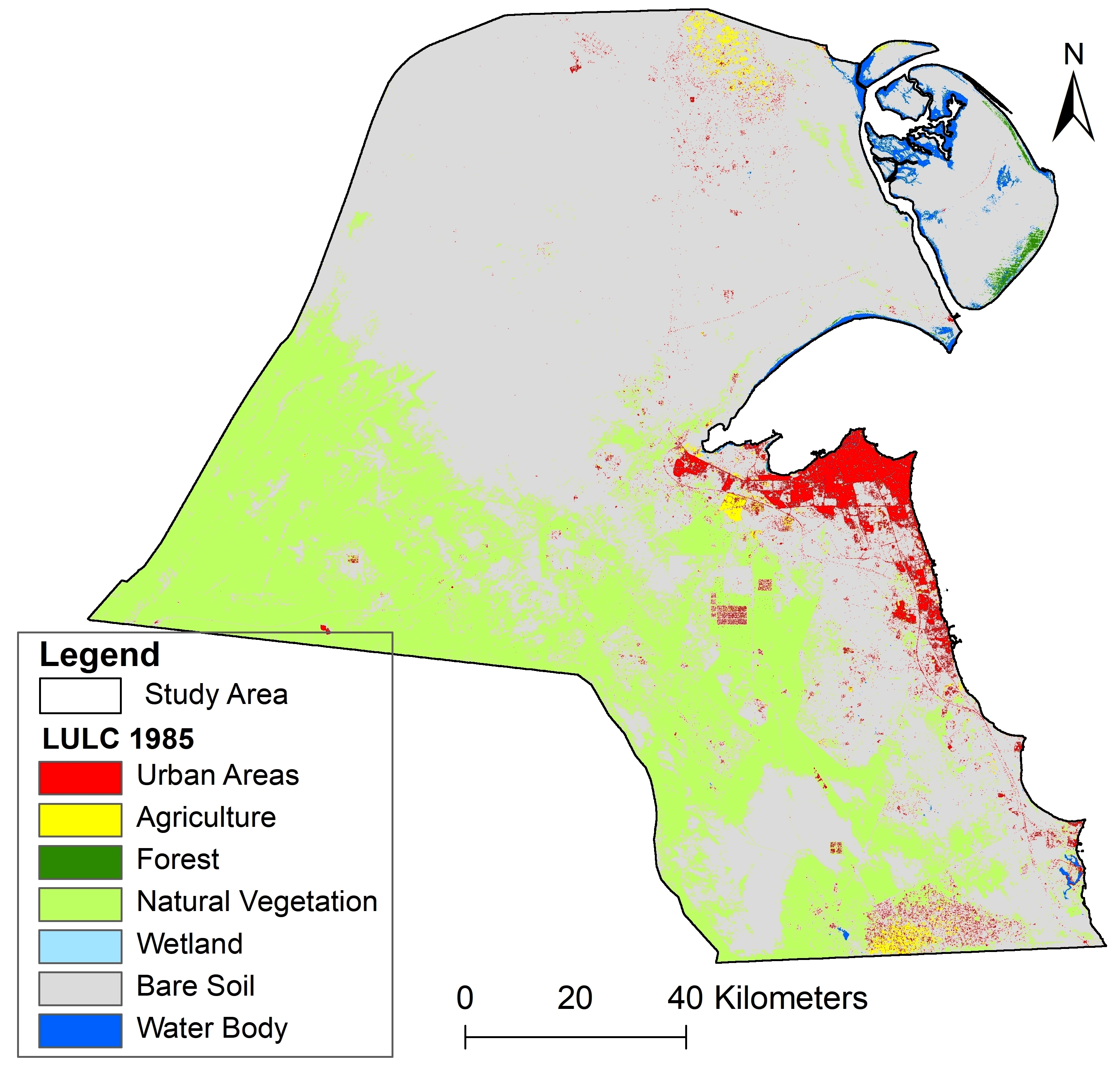 | 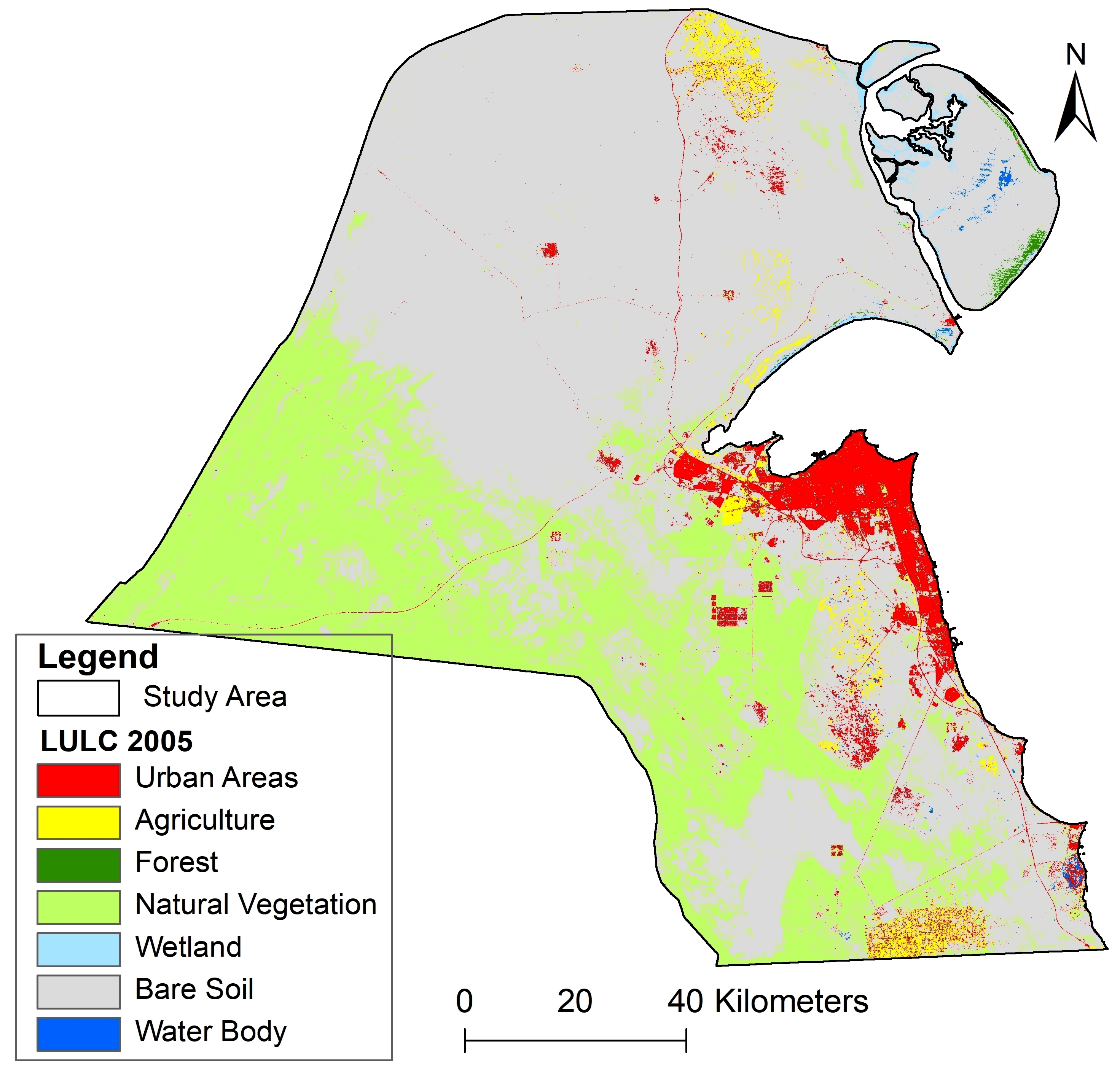 |
| --- | --- |
| **(a)** | **(b)** |
| 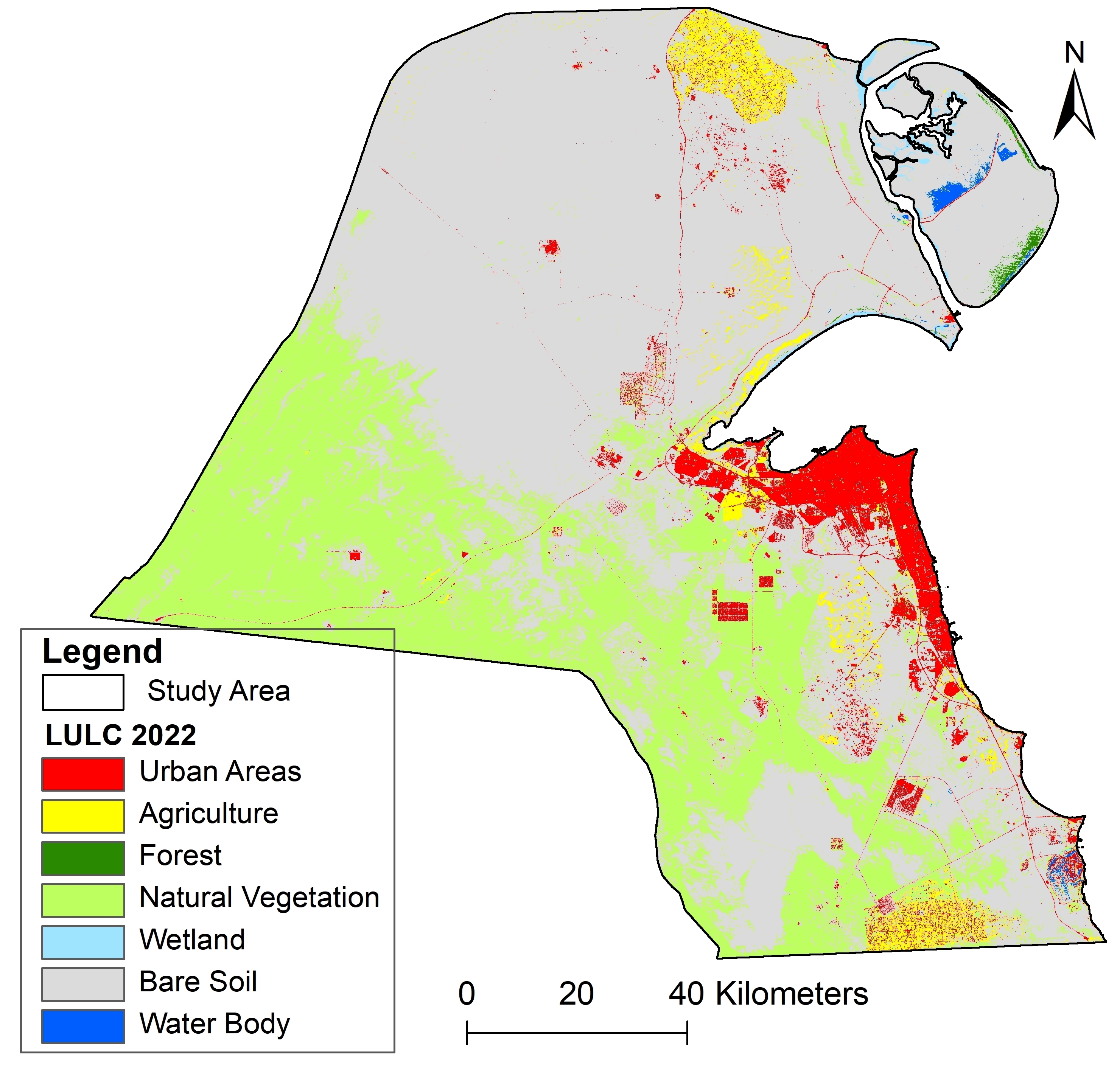 | 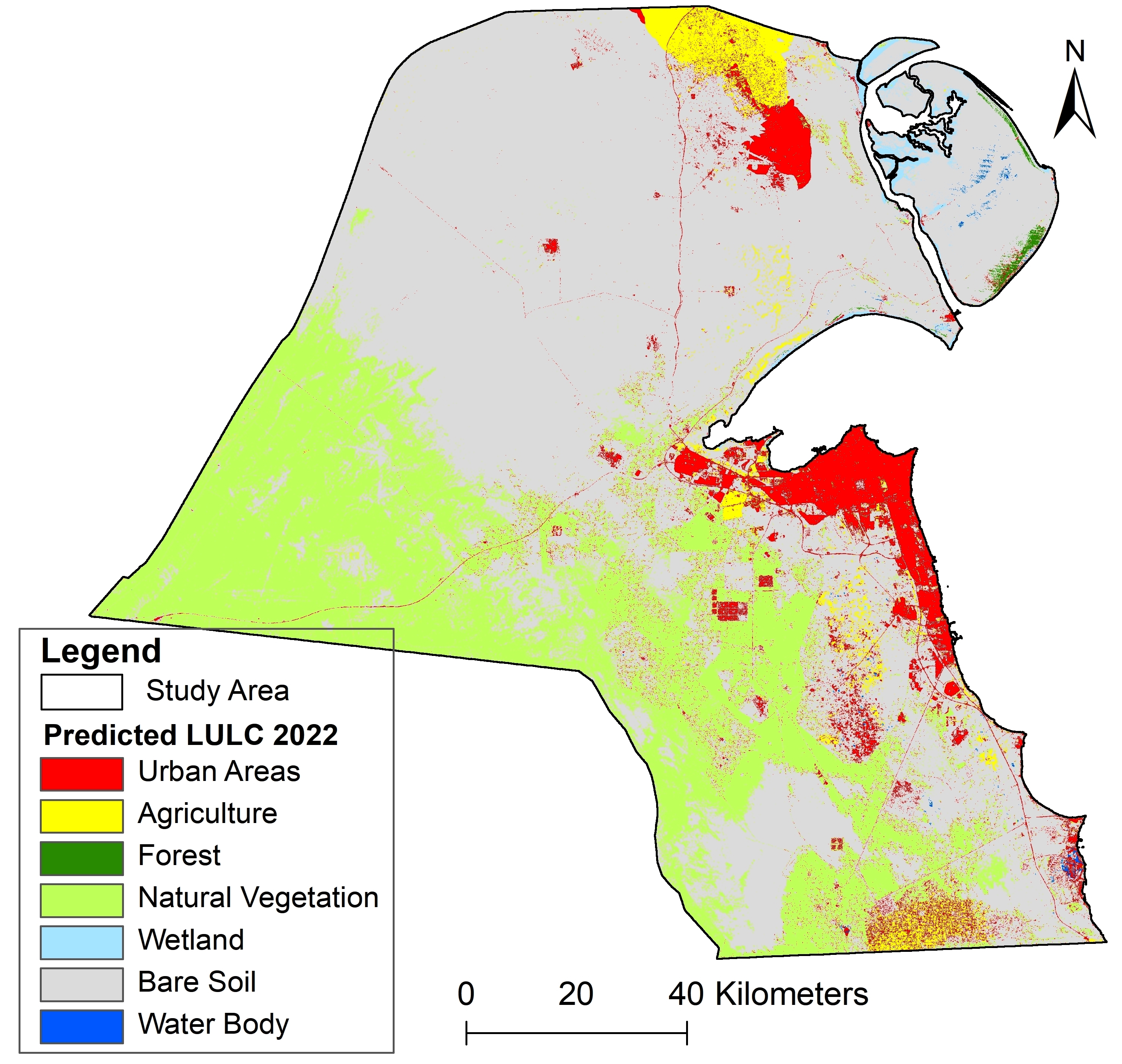 |
| **(c)** | **(d)** |

**Fig. S4** Actual LULC maps for the years [75,76] (a) 1985 (b) 2005 (c) 2022 and (d) the predicted LULC map for the year 2022

Supplement: S4 Fig — (DOCX) [file pone.0318604.s008.docx]
